# Whole-Genome Analysis Reveals That Active Heat Shock Factor Binding Sites Are Mostly Associated with Non-Heat Shock Genes in *Drosophila melanogaster*

**DOI:** 10.1371/journal.pone.0015934

**Published:** 2011-01-14

**Authors:** Sarah E. Gonsalves, Alan M. Moses, Zak Razak, Francois Robert, J. Timothy Westwood

**Affiliations:** 1 Department of Cell and Systems Biology, University of Toronto, Mississauga, Canada; 2 Department of Cell and Systems Biology, University of Toronto, Toronto, Canada; 3 Centre for the Analysis of Genome Evolution and Function, University of Toronto, Toronto, Canada; 4 Institut de Recherches Cliniques de Montréal, Montréal, Canada; 5 Département de Médecine, Faculté de Médecine, Université de Montréal, Montréal, Canada; National Cancer Institute, United States of America

## Abstract

During heat shock (HS) and other stresses, HS gene transcription in eukaryotes is up-regulated by the transcription factor heat shock factor (HSF). While the identities of the major HS genes have been known for more than 30 years, it has been suspected that HSF binds to numerous other genes and potentially regulates their transcription. In this study, we have used a chromatin immunoprecipitation and microarray (ChIP-chip) approach to identify 434 regions in the *Drosophila* genome that are bound by HSF. We have also performed a transcript analysis of heat shocked Kc167 cells and third instar larvae and compared them to HSF binding sites. The heat-induced transcription profiles were quite different between cells and larvae and surprisingly only about 10% of the genes associated with HSF binding sites show changed transcription. There were also genes that showed changes in transcript levels that did not appear to correlate with HSF binding sites. Analysis of the locations of the HSF binding sites revealed that 57% were contained within genes with approximately 2/3rds of these sites being in introns. We also found that the insulator protein, BEAF, has enriched binding prior to HS to promoters of genes that are bound by HSF upon HS but that are not transcriptionally induced during HS. When the genes associated with HSF binding sites in promoters were analyzed for gene ontology terms, categories such as stress response and transferase activity were enriched whereas analysis of genes having HSF binding sites in introns identified those categories plus ones related to developmental processes and reproduction. These results suggest that *Drosophila* HSF may be regulating many genes besides the known HS genes and that some of these genes may be regulated during non-stress conditions.

## Introduction

More than four decades ago Ritossa described a phenomenon where specific loci on the polytene chromosomes from third instar larvae of *Drosophila* decondensed or “puffed” when the larvae were exposed to heat or other forms of stress such as oxidative stress, inhibitors of respiration and certain metals [1]. These puffs represented heat-induced sites of gene transcription and the genes residing there became known as the heat shock (HS) genes and their protein products the heat shock proteins (HSPs). The stress induced molecular and cellular events collectively became known as the heat shock response and is highly conserved in all organisms. During normal and stressed conditions, HSPs and their cognate proteins (HSCs) have essential functions in helping proteins fold properly, acting as protein chaperones during protein synthesis, processing, and degradation as well as the translocation of proteins across intracellular membranes [2], [3]. HSPs are also known to have direct and important positive functions in a number of disease conditions and pathophysiological states including immunity against infection, ischemia, neural injury, and neural degenerative diseases [4].

Heat shock gene regulation in eukaryotes occurs at the transcriptional and post-transcriptional levels. Stress induced HS gene transcription is governed by the protein factor called Heat Shock Factor (HSF). HSF recognizes and binds to a specific DNA sequence in the promoter of HS genes known as the HS element (HSE) [5], [6], [7] (for a review of HSEs see [8]). Single genes for HSF have been cloned from yeast, fruit flies (*Drosophila*), and frogs, and multiple homologous but distinct HSF genes have been cloned in chickens, mice, and humans. The HSF that is primarily involved in responding to heat and other stress agents has been designated HSF1 in most species with multiple HSFs (for reviews of HSF see [9], [10], [11], [12], [13]). HSF is present in cells at all times and is activated to its transcriptionally competent form upon stress. In the metazoans studied thus far, binding of HSF or HSF1 to HSEs is low to virtually nonexistent in unshocked cells and upon HS or other stresses, HSF converts from a monomer to a trimeric form that binds to the HSEs with high affinity.

HSF is an essential gene in those species that have a single HSF gene (e.g. yeast and *Drosophila*) even under non-stress conditions. In the case of *Drosophila*, death was found to occur between the first and second larval instar stages in null mutants suggesting a critical role for HSF even under non-stress conditions [14]. In addition, the same study found that HSF was required for oogenesis. Furthermore, mice lacking HSF1 can live to adulthood but have a severely compromised stress response and display several other defects including prenatal lethality, growth retardation and female infertility [15]. *Hsf1 ^−/−^* female mice also produce defective oocytes that, when fertilized, do not develop very far into embryogenesis [16]. Mammalian HSF1 and HSF4 play important roles in lens and olfactory epithelium development [17], [18] and a mutation in HSF4 is associated with heritable cataract formation in humans [19]. *Hsf2^−/−^* mice show embryonic brain defects that persist with adults displaying enlarged ventricles and a decrease in hippocampus size and striatum and cortex width [20], [21]. Moreover, both HSF1 and HSF2 play roles in sperm development in mice [20], [21], [22].

There have been a few genome-wide screens using DNA microarrays to characterize the eukaryotic transcriptional response to HS in *C. elegans* [23], human cell lines [24], [25], *Drosophila* embryos [26], and *Drosophila* adults [27]. In addition to standard expression microarray experiments, others have used chromatin immunoprecipitation coupled with microarrays (ChIP-chip) to find HSF binding sites: in yeast, using probes in intergenic and coding regions [28]; in human tissue culture cells for HSF1, using a custom 768 element promoter array [25]; in *Drosophila* embryos using a 5400 element cDNA array and 3000 element tiling array [29]; and in mouse testis for HSF2 using a 26,000 promoter tiling array [30]. There has also been a recent study that has examined the binding sites for HSF in *Drosophila* S2 cells using ChIP and next generation DNA sequencing (ChIP-seq) [31].

When the polytene chromosomes from heat-shocked *Drosophila* 3^rd^ instar larvae were stained with anti-HSF antibodies, HSF was found to be localized to more than 200 loci [32]. Given that only nine well documented HS gene loci existed at the time, the authors proposed that HSF had additional genomic targets besides the well known major HS genes, perhaps stimulating lesser known HSP and HSC genes, other “novel” heat-induced genes. In addition, it was hypothesized that *Drosophila* HSF might also play a role in the transcriptional repression of certain other genes that are known to be repressed during HS. Supporting this hypothesis, HSF1 in human cells has been shown to be a repressor of cytokine genes [33]. In this study we have identified more precisely, using ChIP-chip analysis with genome-tiling arrays, more than 430 HSF binding sites in the *Drosophila* genome. We have also performed transcription analysis of heat shocked Kc167 cells and 3^rd^ instar larvae in an attempt to correlate HSF binding events with induction of gene transcription.

## Results

### Identification of HSF binding sites in the *Drosophila* genome

**Figure 1:**
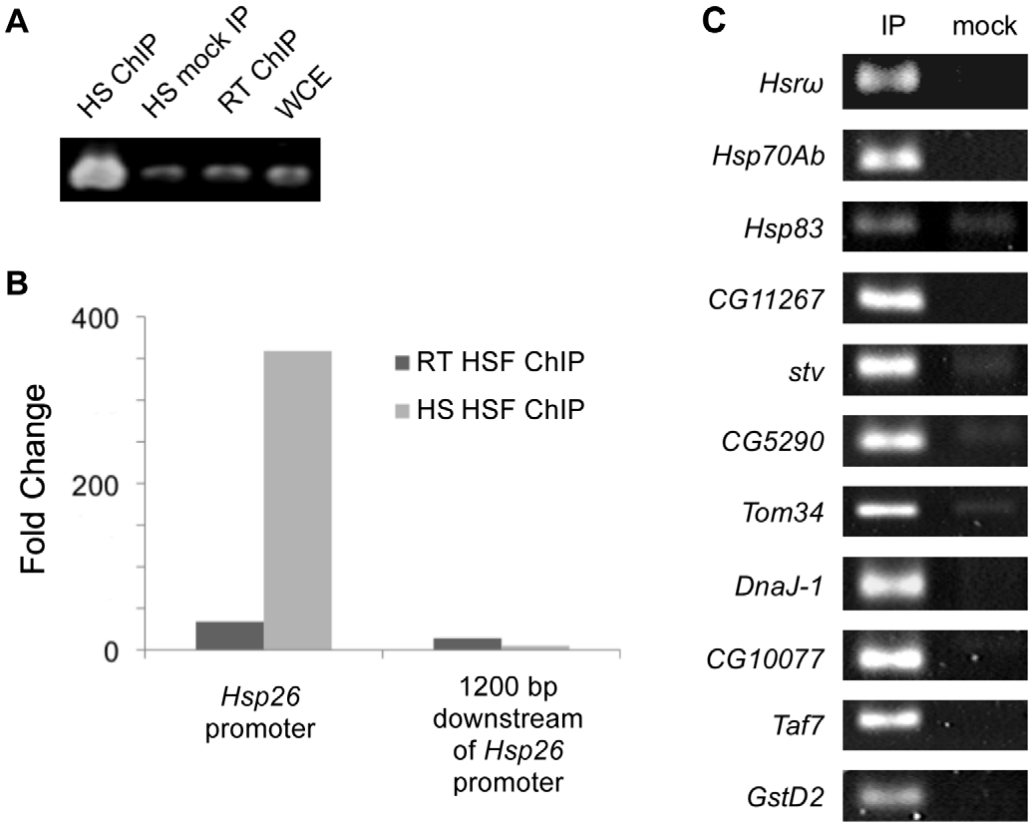
Confirmation of HSF binding to select regions. (A) Enrichment of the region upstream of the *Hsp26* gene by HSF ChIP following heat shock (HS; 36.5°C, lane 1) relative to whole cell extract (WCE; lane 4), HSF ChIP at room temperature (RT; 22°C, lane 3) and no antibody mock ChIP following HS (lane 2) by end-point PCR. (B) qPCR confirmation of enrichment of the same region as in (A) by HSF ChIP following HS (light grey) relative to HSF ChIP at RT (dark grey) and of a region 1200 bp downstream of the *Hsp26* gene. (C) PCR amplification of select regions associated with the genes indicated on both an anti-HSF IP enriched sample (left column) and mock IP sample (right column).

We performed ChIP-chip analysis on heat-shocked Kc167 [34] to identify HSF binding sites across the *Drosophila* genome (data available in GEO under GSE19744). HSF binding should reach a maximum level following a 30-minute heat-shock (HS) at 36.5°C [35], [36] so we conducted our heat-shock treatment under this condition. We fixed both heat-shocked and non-shocked cells with formaldehyde to preserve protein-DNA interactions and then immunoprecipitated HSF bound chromatin complexes with an anti-HSF antibody generated by Westwood et al.,[32]. This antibody is specific for HSF and has been used to visualize HSF binding sites at over 200 loci in *Drosophila* polytene chromosomes by indirect immunofluorescence [32]. As a control for non-specific binding and for HSF binding under non-shock conditions, we also performed a mock ChIP without antibody and an anti-HSF ChIP at room temperature ((RT), 22°C) respectively. We confirmed that our ChIP had successfully enriched for HSF-bound chromatin in HS cells but not mock treated or RT cells by measuring the relative abundance of *Hsp26* promoter DNA by PCR and qPCR (Figure 1). Furthermore, we confirmed that our ChIP conditions were specific enough that we did not get enrichment of the sequence 1200bp downstream of the *Hsp26* promoter (Figure 1B). Following confirmation, we amplified fragments from two independently produced HS anti-HSF ChIP, RT anti-HSF ChIP, and mock ChIP samples by ligation mediated-PCR, labeled them with fluorescent dyes, and hybridized them to genome tiling arrays (Agilent Technologies) as described in the Methods section.

Agilent's Feature Extraction software quantified images of the arrays and Agilent's ChIP Analytics software identified probes corresponding to regions or segments of chromatin that were bound by HSF. We compared the anti-HSF and mock ChIP segments to determine if any of the anti-HSF ChIP segments were non-specific. Only two ChIP segments exhibited any degree of overlap to mock ChIP segments: the first (chr3R:11,071,788-11,074,349) only partially overlaps with a mock ChIP segment and exhibits a much stronger min P[Xbar] on the ChIP array (1.16×10^−11^ vs 6.36×10^−4^). Furthermore, this segment is contained in one of the loci (88E) bound by HSF on polytene chromosomes [32] and so was retained as part of the dataset. The second segment (chr3L:18,124,038-18,125,011) completely overlaps with a mock ChIP segment and has a comparable min P[Xbar] to that of the mock ChIP segment, therefore, this segment was omitted from further analysis.

**Figure 2:**
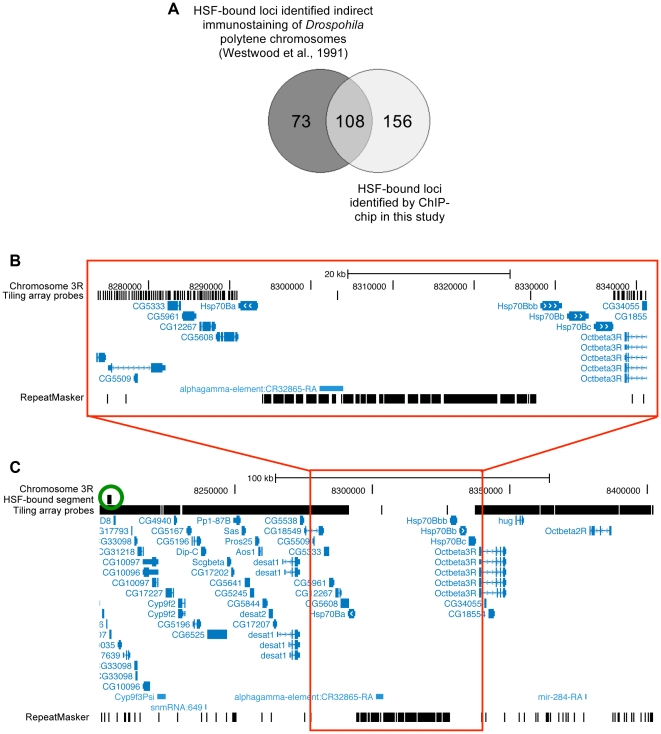
Overlap of HSF binding sites indentified by ChIP-chip of Kc cells and immunostaining of polytene chromosomes of 3^rd^ instar larvae. (A) The 434 HSF-bound sites indentified by ChIP-chip analysis were mapped to 265 unique cytolocations for this comparison. (B) Zoomed-in view of the region of chromosome 3R where the *Hsp70B* genes are located highlighting both the absence of tiling-array probes and the repetitive and/or low complexity sequence in this region as indicated by the RepeatMasker track (bottom). (C) Expanded view of (B) to show the location of the nearest HSF-bound (green circle). This image, generated using the UCSC Genome Browser, illustrates the chromosome region represented in bp (according to release 4.2 of the *Drosophila* genome) as indicated at the top. Genes are depicted as blue boxes with the thick and thin parts representing exons and introns respectively. Arrows (either blue or white) within the gene indicate the direction of transcription. Vertical black lines show the location of each probe on the Agilent 2×244k tiling arrays (Agilent Technologies) in the depicted region.

**Figure 3:**
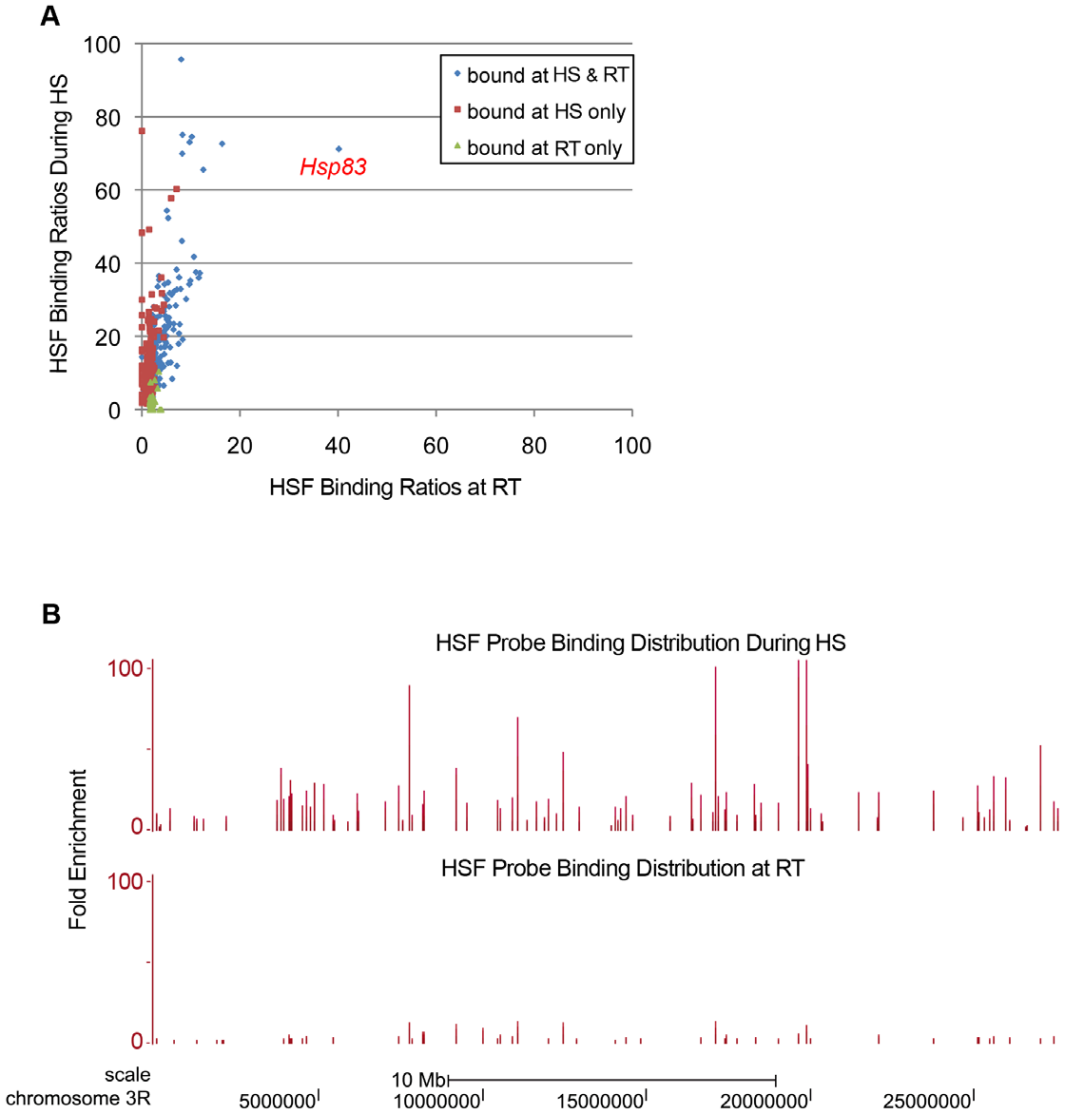
Comparison of HSF binding under HS and non-HS (RT) conditions. (A) Scatter plot of the HSF binding ratio of representative probes from segments bound by HSF during both HS and RT (blue diamonds), during HS only (red squares), or at RT only (green triangles). Although most of the sites bound by HSF at RT overlap with sites bound during HS, levels of HSF binding are greatly diminished at RT (blue diamonds). As expected the only region to be strongly bound by HSF at RT is the region upstream of *Hsp83*. (B) Level of HSF binding during HS (top) and at RT (bottom) to chromosome 3R. Each bar represents a probe from the tiling array that is part of an HSF bound segment and its height indicates its fold enrichment relative to WCE.

In total we identified 434 HSF bound chromatin segments including regions associated with all but one of the known major heat-inducible genes. A selection of targets was confirmed by PCR (Figure 1C). No HSF binding site was detected upstream of any of the *Hsp70B* genes due to the absence of probes on the tiling array in this highly repetitive region of genome (Figure 2B,C). We next examined regions bound by HSF at RT (non-HS conditions) and found that 81% coincided with segments bound by HSF under HS conditions, however, in every case, the level of HSF binding is substantially less at RT (i.e. 5-fold lower on average) (Figure 3 and Table S1; data also available in GEO under GSE22335). The most highly enriched HSF binding site at RT is located upstream of *Hsp83* in one of the only regions specifically occupied by HSF under non-HS conditions [36]. With the possible exception of this site, the observed weak HSF binding in the RT samples may reflect transient HSF binding, HSF binding in a subset of cells, and/or is the result of the induction of a mild HS response brought on by the initial harvesting and fixation of the cells.

Table 1 lists all sites bound by HSF during HS treatment with a greater than 30-fold enrichment over whole cell extract (WCE) including all the sites associated with the major heat-inducible genes. Where applicable, the staining intensity observed on polytene chromosomes by Westwood et al.,[32] is indicated. Twenty-nine of our top 40 sites map to 24 loci that overlap with HSF-bound loci in polytene chromosomes. When we consider our entire set of HSF-bound sites the overlap with the polytene data is significant (p-value from X^2^ test  = 2.5×10^−10^; Figure 2A). Of those 73 loci identified by Westwood et al.,[32] that did not overlap directly with our ChIP-chip data, we found that 54 are within one cytological band of at least one HSF binding site (data not shown). Such an offset is within the estimated error rate associated with computing cytological locations based on sequence location (Flybase Reference Manual G, section G.5.1 [37]). If we consider these 54 sites offset by one cytological band, together with the 108 that directly overlap, then 90% of the HSF-bound loci identified on polytene chromosomes are covered in our ChIP-chip data. A X^2^ test on the independence of the two datasets taking into account the offset indicates that there is overlap between them (p-value from X^2^ test  = 4.5×10^−6^).

**Table 1:** Chromatin segments bound by HSF exhibiting a 30 or greater fold enrichment over whole cell extract (WCE).

HSF binding site						closest gene(s)		
location of peak of the HSF bound segment	fold change	P[Xbar]	HSE (p-val<1×10^−4^) location relative to peak (bp)	cytology	polytene staining intensity (Westwood et al 1991)	name	position of peak of binding site relative to gene TSS (bp)	location of peak of binding site
3R:17121946..17122005	96	1.10E-07	98	93D^1^		*Hsrω*	−368	Intergenic
2L:13165433..13165492	76	3.70E-11	−161	34A	1	*Sir2 & DnaJ-H*	−532	Intergenic
3R:19883079..19883127	75	6.80E-08	25	95D	2.5	*Hsp68*	−74	Intergenic
3L:9351650..9351707	75	8.80E-08	0	67B	4	*Hsp26 & Hsp67Ba*	−3051707	Intergenic
3R:7783512..7783571	73	2.00E-10	−447	87A	3	*Hsp70Ab & Hsp70Aa*	−1760	Intergenic
2L:295186..295245	73	3.20E-09	74	21B	1	*Hop*	108	Exon1
3L:3176534..3176591	71	7.60E-08	−102	63B	3	*Hsp83*	5	Exon1
3L:9357629..9357688	70	6.70E-08	−3	67B	4	*Hsp27 & Hsp23*	−3521829	Intergenic
3R:11068766..11068825	65	3.60E-12	22	88E	1.5	*Hsc70-4*	−81	Intergenic
3L:22008546..22008605	60	1.60E-05	−51	79B		*CG7133*	−213	Intergenic
3L:9347685..9347744	58	1.90E-04	−142	67B	4	*Hsp22 & Hsp67Bb*	−249838	Intergenic
X:20846528..20846587	54	1.20E-06	−123	19E		*Ntf-2*	409	Intron1
X:5725222..5725281	52	1.00E-07	−62	5C	2	*CG16721*	159	Exon1
3R:27045350..27045409	49	1.70E-07	NA	100B	1	*CG1746*	−23	Intergenic
3L:16745454..16745513	48	2.10E-07	133	73C	1	*CG9705*	115	Exon1
2L:22342021..22342080	46	2.20E-11	135	40F	1.5	*CG17018*	42528	Intron1
X:10954037..10954096	42	2.30E-07	77	10A	1	*Hsp60*	377	Intron1
3L:12990240..12990299	38	2.00E-07	88	69F		*CG11267*	267	Intron1
2L:6966386..6966445	38	9.80E-12	-25	27C		*smt3*	1177	downstream
3R:12473487..12473539	37	7.30E-12	15	89D	1	*Cctgamma*	102	Exon1
3L:13454266..13454312	36	5.50E-07	3	70B		*stv*	−101	Intergenic
3L:3886074..3886133	36	2.40E-07	32	63F		*Ubi-p63E*	348	Exon2
2L:5009864..5009923	36	2.40E-08	−16	25C	1	*Rtnl1*	−150	Intergenic
3R:9208591..9208650	36	2.60E-11	107	87E	2	*Droj2*	78	Exon1
2L:12046460..12046519	35	2.40E-11	218	33B	3	*CG6770*	−390	Intergenic
3R:11072836..11072895	35	1.20E-11	17	88E	1.5	*Hsc70-4*	2889	downstream
3L:7839477..7839536	35	6.10E-07	−6	66A		*Pdp1*	1812	Intron1
3L:17877705..17877764	34	4.30E-07	−30	75A	3	*CG5290*	81	Exon1
3L:8492559..8492618	34	1.00E-06	186	66D	2	*CG6776*	−245	Intergenic
X:11204481..11204540	34	2.50E-06	48	10B	2	*CG11750*	57	Exon1
X:2503300..2503359	33	1.60E-07	6	3A	1	*sgg*	2182	Intron1
X:6499517..6499576	33	1.30E-07	106	6C		*CG3226*	−125	Intergenic
2L:22157037..22157096	32	2.40E-11	NA	40F	1.5	*CG1832*	−313	Intergenic
U:5800980..5801039	32	1.20E-07	40	53F	0.5	*CAP*	1331	Intron2
3R:25608959..25609018	32	6.10E-07	13	99C		*kay*	657	Intron1
3R:3859259..3859318	31	4.50E-11	89	84E	2	*Tom34 & CG11035*	263 & −620	Intron1
3L:112600..112659	31	2.40E-07	16	61B		*Pk61C*	−353	Intergenic
3L:8678764..8678823	31	3.10E-07	86	66D	2			
3L:14002678..14002737	30	1.30E-07	171	70C	1	*Hsc70Cb*	195	Exon1
2L:16517060..16517119	30	8.20E-11	NA	36A	1.5	*CG5953*	11716	Intron2
3L:19838272..19838331	30	1.80E-06	−31	76D		*Su(Tpl)*	5748	Intron1

1 While no staining of HSF was noted on polytene chromosomes at 93D, a staining intensity of 1.5 was observed at 93C.

### Heat Shock Elements (HSEs)

**Figure 4:**
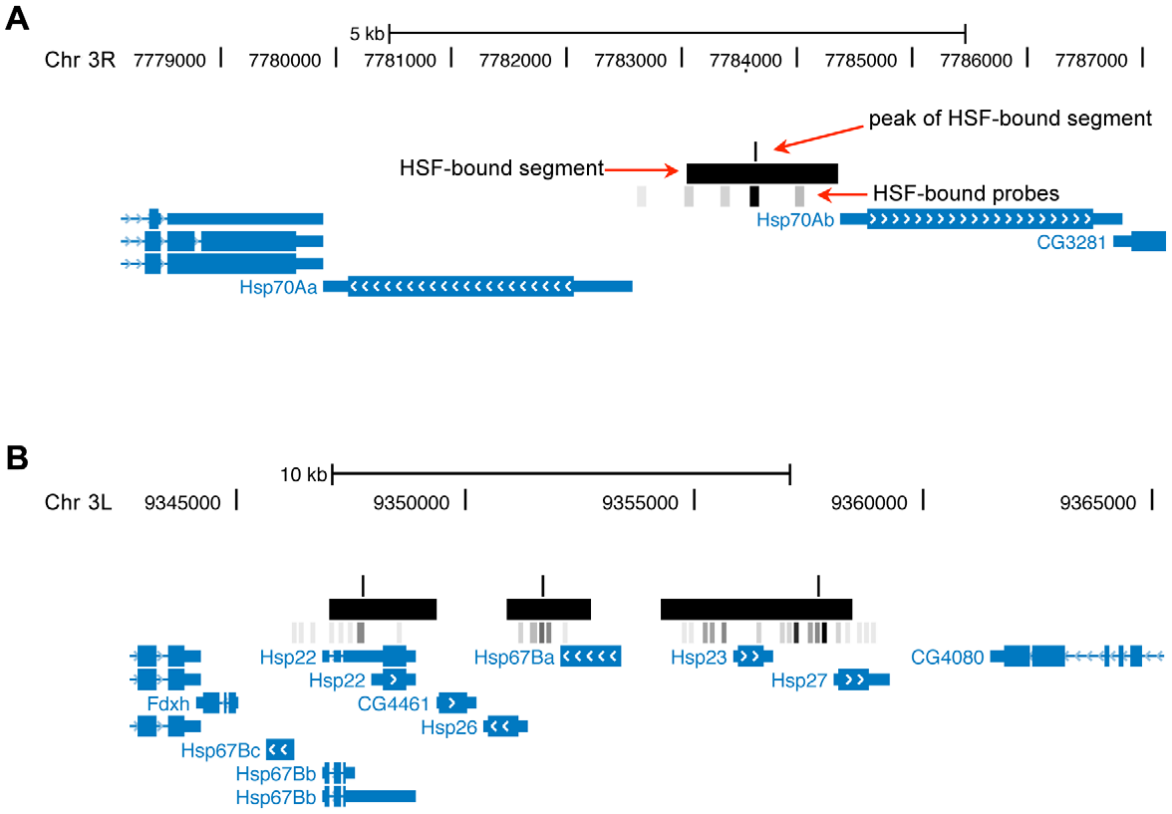
Representation of select genomic regions bound by HSF. This image, generated using the UCSC Genome Browser, illustrates the chromosome region represented in bp (according to release 4.2 of the *Drosophila* genome) as indicated at the top. Genes are depicted as blue boxes with the thick and thin parts representing exons and introns respectively. Arrows (either blue or white) within the gene indicate the direction of transcription. Large black and small grey boxes represent HSF-bound segments and probes respectively identified by Chip Analytics (Agilent Technologies). Darker grey shading is used to represent probes with higher fold-enrichment relative to WCE. The single black line above bound segments indicate the position assigned as the segment peak and is also the center of the probe with the lowest P[Xbar]. (A) A single HSF-bound segment is found in the region encompassing *Hsp70A*. The probe with the lowest P[Xbar] is located near the numerical center of the bound segment. (B) Three HSF-bound segments are found in the region encompassing the small HSP genes. It is likely that these segments represent more than 3 distinct HSF-binding sites, however, a single peak per segment has been assigned potentially resulting in an underestimation of the number of individual binding sites in this region.

**Figure 5:**
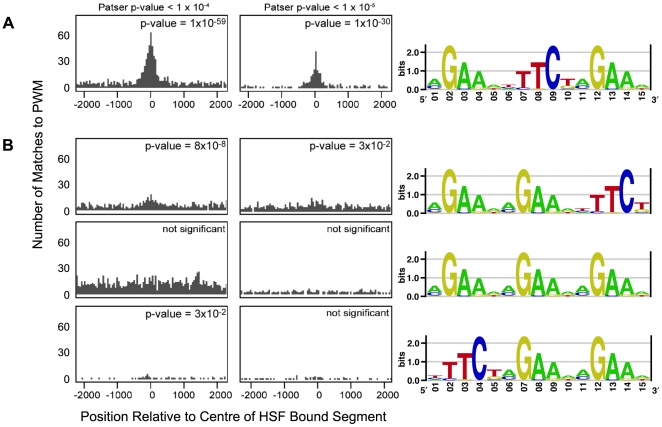
The 5 bp motif nGAAn arranged as direct inverted repeats is enriched in HSF-bound segments. We used the pattern-matching program Patser [38] to score the occurrences of 4 PWMs from TRANSFAC (M00165, M00164, M00167, M00166), depicted as sequence logos on the right side of the figure, in both the sequence bound by HSF and the local background (up to 2500 bp on either side of the peak of each HSF binding site). The histograms show the number of matches to each PWM in 50 bp windows centered on the peak of each HSF-bound segment. P-values at the top of each column of graphs indicate the cut-off used when considering a match by Patser. P-values at the top right of each histogram indicate the probability given by a X^2^ test that the difference in the number of matches to the PWM found in the sequence at the peak and in the local background is due to chance. (A) The motif nGAAnnTTCnnGAAn shows significant enrichment at the peak of the HSF-bound regions at both Patser p-value cut-offs (compare left and right columns) and occurs more frequently than any other orientation of this motif in HSF-bound regions (compare A and B). (B) Other orientations of the 5 bp core motif not as significantly enriched as the motif in (A), if at all, in the HSF-bound segments. What little enrichment is seen for the alternate orientations when the Patser p-value cut-off is set to <1×10^−4^ (left column) is essentially lost when the Patser p-value cut-off is lowered to consider only highly probable matches (p-value<1×10^−5^, right column). Sequence logos were generated from TRANSFAC PWMs M00165, M00164, M00167, M00166 using the online web tool enoLOGOS [72].

The HSF bound segments identified in our analysis span several oligonuclotide probes from the tiling array and average 1400 bp in length. Thus, for each HSF bound segment we assigned a “peak” as the center of probe in the segment with the lowest P[Xbar] (i.e. the probe with the lowest probability that the observed difference between ChIP and WCE signals is due to non-biological causes). An example of how a peak identified in this way compares to the HSF-bound segments and probes identified by ChIP Analytics is presented in Figure 4. Figure 4A depicts the typical scenario where the HSF-bound probe with the lowest P[Xbar] in a given segment also exhibits the highest fold change relative to WCE in that segment and is approximately in the center of that segment. In the situation where clusters of HSF-bound sites are found over small distances, assigning a single peak to segments that may represent more than one binding site may result in an underestimation of the total number of true sites. This is the case for the HSP gene dense region on chromosome 3L where there is no peak for the HSF-binding site upstream of *Hsp23* because it is incorporated into the neighboring segment due to its proximity (Figure 4B). Using Patser [38], we scanned 2500 bp of sequence flanking each peak to find matches to the position weight matrix (PWM) representing the canonical 15 bp HSE from TRANSFAC (Figure 5A). A X^2^ test revealed a significant difference in the number of matches to this motif between the sequence around the peak and the flanking sequence (p-value = 1×10^−59^, Figure 5A) suggesting that assigning the peak as stated above was reasonable. This analysis also suggested that the most HSE rich region lies between −400 bp to +300 bp of each assigned peak (Figure 5A). The position of the HSE (p-value <1×10^−4^) closest to the peak of each binding site is listed in Table 1 and in Table S2.

Because the canonical HSE is composed of 3 repeats of the 5 bp motif nGAAn we sought to determine if other orientations of this motif were enriched in the peak region relative to the local background. We used Patser to determine the number of matches to each PWM for all possible 3-way combinations of the 5 bp motif represented in TRANSFAC and plotted a histogram to depict the distribution of matches (Figure 5B). Two of the alternate orientations are slightly enriched near the center of the fragments although not as strongly as the canonical motif (compare left columns of Figure 5A and Figure 5B nGAAnnGAAnnTTCn and nTTCnnGAAnnGAAn). When the stringency of what may be considered a “match” to the PWM was increased (ie. by decreasing the Paster p-value from <1×10^−4^ to 1×10^−5^, right columns of Figure 5B), the number of matches to these alternate motifs were however, essentially reduced to background levels, while the total number of matches to the canonical HSE was still significantly above background levels (p-value = 1×10^−30^, right column of Figure 5A). Taken together, the result of this analysis suggests that the inverted repeat arrangement is strongly favored over all of the orientations examined.

### Genes associated with HSF binding events

Because the previous analysis of HSF binding events on polytene chromosomes was not of sufficient resolution to determine which genes HSF associated with at heat shock temperatures on a global scale, we set out to determine what genes may be affected by HSF binding by identifying the genes closest to each HSF binding site. In a first attempt to identify genes that may be regulated by HSF binding, we identified the nearest transcription start site (TSS) to the peak of each HSF bound segment. The result of this analysis is included in Table 1 for the most strongly bound sites and in Table S2 for these and all the remaining sites. Since we cannot rule out the possibility that HSF may be acting on more distant genes, we extended this analysis to identify all genes within a 2500 bp window centered on the peak of each HSF-bound segment and calculated the distance from peak to TSS for each of these genes. For any given binding site, there may be more than one gene within the 2500 bp window so Table S2 lists all genes found within the window in order of proximity to the binding site.

**Figure 6:**
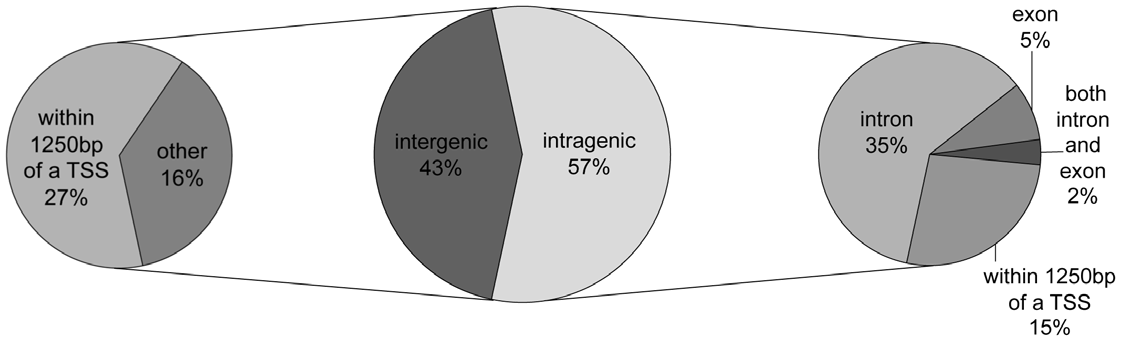
Breakdown of HSF-binding site by location. Fewer than 1/3 of all HSF-binding sites are located in gene promoters with the promoters of major heat-inducible genes accounting for less than 5% of these sites (not shown). The majority of HSF binding sites are instead located in transcribed regions of the genome with introns accounting for the largest proportion of HSF targets. Fifteen percent of all sites are somewhat ambiguous in definition as they occur within transcribed regions but are also in the vicinity of a transcription start site (TSS).

**Figure 7:**
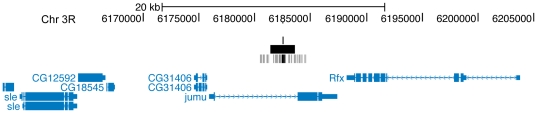
Example of an intronic HSF-binding site. As with Figure 4, this image was generated using the UCSC Genome Browser to illustrate the chromosome region represented in bp (according to release 4.2 of the *Drosophila* genome) as indicated at the top and the legend is the same as that in Figure 4.

In the course of this analysis, it became apparent that there were instances in which HSF was binding within the transcribed region of many genes. As such, we investigated the proportion of sites that were found within transcribed regions (intragenic) relative to those that were not (intergenic) (Figure 6). In total, 57% of all sites were located in the transcribed region of at least one gene with a preference for binding within introns (Figure 6). In contrast, only 41% of euchromatic sequence is intragenic. An example of HSF-binding within an intron is presented in Figure 7 for the transcription factor *jumu*. Roughly 1/4 of sites in transcribed regions were, however, also located in the proximity of gene promoters (which we are considering to be a region surrounding 1250 bp from the transcription start site). By our definition, 14% of the genome falls in gene promoters, however, of the 43% of sites that are intergenic, over half were found in promoter regions representing 27% of all HSF binding sites (Figure 6). It should be noted that the promoters of the major HSP genes account for less than 5% of all HSF bound promoters.

**Figure 8:**
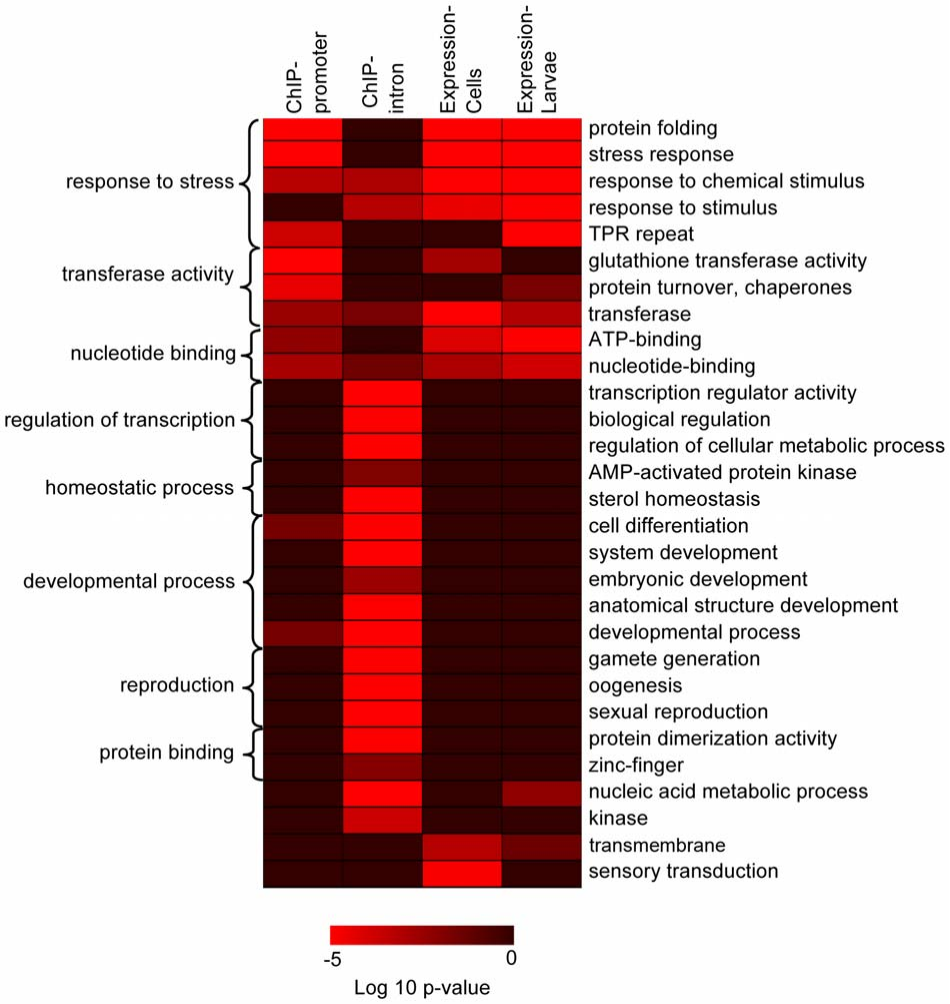
Heat map summary of select categories from DAVID functional enrichment analysis. Genes that were associated with HSF-binding sites in either the promoter or intron regions (column 1 and 2 respectively) and genes that were differentially regulated by HS treatment in either Kc cells or 3^rd^ instar larvae (columns 3 and 4 respectively) were analyzed for functional enrichment. The Functional Annotation Chart tool was used to obtain the p-values describing the probability that a functional term is enriched among genes in the groups examined by random chance, and the Functional Annotation Clustering tool was used to group similar annotation terms. Lower p-values indicating enrichment for the term on the right of the chart are colored in red, while p -values above 0.1 are indicated in maroon. Groups of similar annotation terms are indicated on the left of the chart.

Given this distribution we were interested in determining if HSF is targeting a specific class of genes when binding to promoters so we used the online resource DAVID [39] to assess enrichment in gene function among these genes. For this analysis we considered only those 27% of sites that were within promoter regions as we defined as 1250 bp from a transcription start site and otherwise not within the transcribed region of any gene. Not surprisingly, the most strongly enriched categories were related to the response to stress (Figure 8 first column). Also among the most highly enriched categories was glutathione transferase activity and TPR repeat.

To further this analysis, we investigated the enrichment in functional categories among genes that contained at least one HSF binding site within their transcribed region. In this case we considered only those 35% of sites that were within introns and greater than 1250 bp away from the transcription start site of any gene/isoform. This conservative estimate of the number of HSF binding sites found in introns still represents a 2-fold enrichment over the background distribution since only 17.1% of euchromatin is intronic. Our findings here were largely unexpected; there was a strong enrichment for genes involved in biological regulation and more specifically the regulation of transcription and metabolic processes as well as for genes involved in reproduction and development such as gamete generation and anatomical structure development (Figure 8 second column).

**Figure 9:**
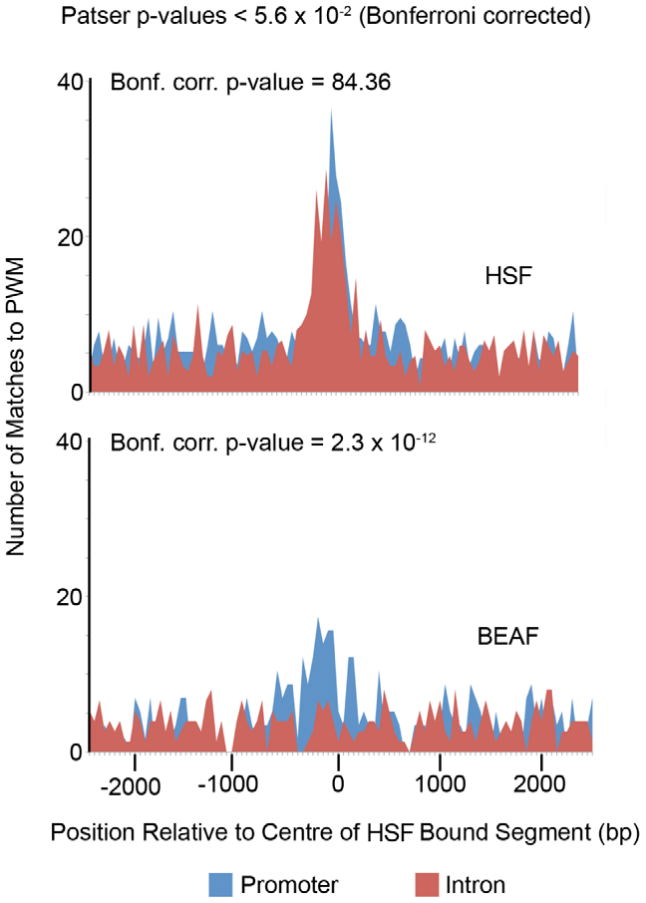
The BEAF *cis* regulatory motif is enriched in promoters, but not introns, bound by HSF. The occurrence of 111 PWMs from TRANSFAC and the *Drosophila* DNase I Footprint Database in HSF-bound promoters and introns was scored with Patser [38]. The histograms show the number of matches (Bonferroni corrected p-value<5.6×10^−2^) to PWMs representing HSF and BEAF binding sites in 50 bp windows centered on the peak of each HSF-bound segment (blue for promoters, red for introns). The PMW for DREF is very similar to the one for BEAF and gives the same result. P-values at the top right of each histogram indicate the probability given by a X^2^ test that the difference in the number of matches to the PWM found in promoters and introns is due to chance.

Because of the difference in functional classification of genes associated with HSF-bound promoters versus HSF-bound introns, we were interested in determining if any other transcription factor(s)/DNA binding protein(s) were associated with these sites. To identify possible candidates, we used Patser to scan HSF-bound promoters and introns for matches (Bonferroni corrected p-value<5.6×10^−2^) to PWMs representing 111 different DNA binding proteins from two databases, Transfac and the *Drosophila* DNase I Footprint Database. As expected, the PWM representing the 15 bp HSE composed of inverted repeats of nGAAn (Figure 5A) was enriched near the peak of both HSF-bound promoters and HSF-bound introns (Figure 9). Of the remaining PWMs, the PWM for BEAF was the only one enriched near the peak of HSF-bound promoters to also have a similar chi squared value (X^2^>40) and show the same level of significance in a chi square test (Bonferroni corrected p-value<2.3×10^−8^) as the PWM representing the canonical HSE (Figure 9). Unlike the HSE PWM, however, this enrichment was only seen for those HSF-bound sites found in promoters; the occurrence of the BEAF PWM in HSF-bound segments located within introns was no different than the background (Figure 9). Consistent with this finding is a recent report that BEAF (boundary element associated factor) binding sites are enriched in 5′ UTRs and in the first 200 bp upstream of gene's TSS [40], [41]. Genes having both BEAF and HSF binding sites do not appear to be strongly enriched in any categories that differ from those enriched among all promoters except for a modest enrichment for genes with cell cycle annotation (p-value  = 0.0011; data not shown) which is consistent with the function of genes BEAF has been shown to regulate [42].

### Transcriptional profile of Kc cell and 3^rd^ instar larvae in response to heat shock

Previous studies have predicted that heat activated HSF might be inducing the transcription of genes in addition to the well-known HS genes. After HS, it is known that RNA polymerase II (pol II) relocalizes from several hundred discrete loci on polytene chromosomes to a far smaller number of loci with a large amount of pol II accumulation at the HS puff sites [43], [44]. Pol II can be seen at about 50 loci after a 20 minute HS and co-localizes with a subset of the approximately 200 observed HSF binding sites. In addition, nascent transcripts can be seen to co-localize with pol II (J.P. Paraiso, M. Gibson and J.T. Westwood, unpublished results).

To determine if HSF binding had an effect on any of the genes with which it associated following heat stress in addition to the classical heat shock genes, we examined the transcriptional profile of Kc cells under the same conditions in which the binding sites were identified (30 minute HS at 36.5°C). RNA isolated from HS and untreated cells was reverse transcribed, labeled and hybridized to NimbleGen expression microarrays (data available in GEO under GSE19745). We identified 211 genes that showed at least a 2-fold change in expression due to heat shock with a FDR corrected p-value less than 0.01 (Table S3). Not surprisingly, several major HSP genes were strongly induced including *Hsp70, Hsp68, Hsp27, Hsp26, Hsp23,* and *Hsp22*. In addition, seven other genes exhibited fold changes comparable to the small HSPs: *CG32850, CG12507, SP555, Gr63a, CG8086, CG7509*, and *Ir93a*. Aside from *CG7509*, which is repressed in response to both oxidative stress and ER stress in *Drosophila* [45], none of these genes have been associated with the stress response in *Drosophila*. In general, most genes we identified changing in response to heat shock in Kc cells were up-regulated, and showed only a modest change in transcript levels (less than 4-fold) (Table S3).

DAVID analysis of genes differentially regulated in cells revealed an enrichment in many of the same categories enriched among genes whose promoters were associated with HSF following HS (Figure 8 third column). Also like the DAVID analysis on HSF-bound promoters, several genes with similar function to the major HSPs were identified. Interestingly, the terms transferase, transmemebrane, and sensory transduction are enriched among genes regulated by HS in cells but not among genes that associated with HSF binding sites (Figure 8 compare column 1 and 2 to 3) indicating that there is a specific set of functionally related genes that are regulated by HS but that are not associated with HSF.

Because many novel heat responsive genes were identified in this genome-wide screen, we wanted to determine how far away the nearest HSF binding site was relative to transcription start sites of these genes. Table S3 lists the distance from the TSS of each gene to the nearest HSF-binding site. Surprisingly, these genes exhibited mean and median distances of greater than 100 kb and 50 kb respectively. In some cases this may be explained by the lack of detection of a bona-fide HSF binding site by our approach since, although the Agilent genome-tiling array covers the entire 117 MB euchromatic genome, probes are lacking in areas with highly repetitive sequence or sequence with high homology to other regions. For example, there are no probes on the array covering the region ∼40 kb upstream of *Hsp70Bbb* (the most highly induced gene in Kc cells) likely due to the highly repetitive nature of this sequence (Figure 2B,C). Instead the closest site to *Hsp70Bbb* we identified was greater than 100 kb upstream of its TSS. However, since this case is expected to be the exception rather than the rule, it is unlikely to be the cause of a lack of HSF binding to the promoters of the majority of the genes identified. To rule out the possibility that a secondary transcription factor transcribed in response to heat shock may be controlling the expression of some of these genes, we repeated the gene expression analysis in the presence of the translation inhibitor cycloheximide and found no significant affect on the transcription of any of these genes (data not shown).

Given that cell lines do not always provide an accurate picture of the biological response of whole organisms, and that several sites of gene transcription can be observed in addition to the HS puff sites on polytene chromosomes, we next examined the transcriptional response to heat shock in wandering 3^rd^ instar larvae. As with the cells, the larvae were subjected to a 30 min heat shock at 36.5°C to match the conditions used for HSF binding site identification (data available in GEO under GSE19745). Overall, 237 genes exhibited a 2-fold or greater change in expression and a FDR corrected p-value of less than 0.01 (Table S3). As in cells, the majority of genes show a modest change (less than 4-fold), are mostly up-regulated, and are mostly enriched in the same functional categories as promoter-bound genes (Figure 8, fourth column). Furthermore, the functional terms transferase and transmembrane are enriched among HS regulated genes in larvae suggesting that several HS-regulated genes not associated with HSF binding sites are still related in function (Figure 8 compare columns 3 and 4). Table 2A lists all genes exhibiting a 8-fold or greater induction in either cells or larvae. Comparison of all genes that were heat-responsive in cells and in larvae revealed few genes that were universally regulated by HS. Ninety-two percent of all stress-responsive genes identified were only affected in one system. The remaining 8% of genes that were affected in both systems include all of the major HSP genes (except *Hsp83* and *Hsp67Ba*, which were only induced in larvae), *DnaJ-1*, and 22 other genes, of which 10 have been previously associated with at least one other stress in *Drosophila* (Table 2B). Functional enrichment analysis of HS responsive genes in larvae identified several non-classical HSPs predicted to have similar functions as the classical HSPs. Among them, we have identified HSF binding sites in the promoters of at least eight: *CG11035, CG7130, CG7945*, *Droj2, PEK, Sir2, Tom34,* and *tra.*

**Table 2:** Genes regulated by heat shock.

		Kc167 cells		3rd instar larvae				
A	Gene	HS vs RT fold change	FRD corrected p-value	HS vs RT fold change	FDR corrected p-value	position of closest HSF binding site relative to TSS (bp)	cytology	staining intensity in polytene data (Westwood et al., 1991)
	*Hsp70Bbb*	41.9	2.00E-10	103.3	1.80E-07	−124162	87B^1^	
	*Hsp70Ba*	33.3	1.60E-09	162.4	6.30E-10	−89431	87B^1^	
	*Hsp70Bc*	32.1	1.60E-09	140.2	8.30E-10	−130728	87B^1^	
	*Hsp70Ab*	31.7	3.30E-09	169.9	8.80E-10	−732	87A	3
	*Hsp68*	24.7	2.20E-10	106.8	4.20E-08	74	95D	2.5
	*CG32850*	16.6	6.90E-08	1.2	7.00E-01	716852	102B	
	*Hsp22*	14.1	3.70E-09	227	3.30E-08	838	67B	4
	*Hsp26*	12.2	1.40E-09	28.5	1.10E-04	305	67B	4
	*CG12507*	10.9	1.20E-04	4.5	2.40E-03	−163722	14B	
	*SP555*	10.8	3.70E-04	-1	9.70E-01	−28158	25C	1
	*Gr63a*	10.7	4.00E-08	15.4	2.00E-05	5969	63F	
	*CG8086*	10.6	2.40E-06	1.5	4.60E-01	−87278	28F-29A	
	*Hsp23*	10.2	8.60E-08	8.3	5.90E-03	1829	67B	4
	*CG7509*	9.9	4.10E-08	6.7	1.20E-04	13407	64B	0.75
	*Hsp27*	8.8	7.60E-07	7.5	1.80E-04	−352	67B	4
	*Ir93a*	8.3	1.20E-04	1.5	2.00E-01	#N/A	93A	
	*DnaJ-1*	5.3	3.30E-08	42	2.10E-05	−219	64E	
	*Cp18*	2.1	1.30E-03	55	3.10E-08	−5166	66D	
	*stv*	3.9	3.70E-08	29.4	7.10E-10	−421	70B	
	*se*	3.5	2.90E-03	18.7	4.90E-07	−1909	66D	
	*CG5290*	1	8.60E-01	15.3	6.80E-08	−81	75A	
	*Hsp83*	1.3	2.40E-03	15.1	2.70E-06	5	63B	
	*CG14961*	3.2	7.90E-04	14.5	3.10E-05	−79865	63D	
	*Hsp67Bb*	2.1	7.50E-04	14.1	1.60E-04	838	67B	4
	*CG17352*	2.6	8.60E-04	13.5	9.40E-06	4134	66C	
	*CG7130*	1	9.10E-01	9.3	4.70E-04	−1021	79B	
	*CG3280*	1	9.70E-01	8.9	2.90E-06	90028	67C	2
	*Fdxh*	1.2	1.10E-01	8.4	5.90E-06	2731	67B	4
	*CG15199*	−1.5	2.20E-01	8.3	1.00E-04	−32986	10A	1
	*CG6785*	1.5	1.80E-02	8.1	1.30E-07	1290	33B	3

		Kc167 cells		3rd instar larvae				
B	Gene	HS vs RT fold change	FRD corrected p-value	HS vs RT fold change	FDR corrected p-value	position of closest HSF binding site relative to TSS (bp)	cytology	staining intensity in polytene data (Westwood et al., 1991)
	*CG14937*	2.5	3.60E-06	7	5.10E-06	218089	33A	
	*TwdlF*	3	5.30E-04	5.7	1.10E-03	−31204	82A	
	*spz4*	4.9	6.20E-05	5.3	1.60E-03	506289	32F	
	*CG13035*	2.4	5.30E-04	5.1	1.50E-06	96856	72E	
	*CG11619*	2.7	3.10E-04	4.3	3.10E-03	104557	75F	1
	*Acp98AB*	3	5.30E-04	4.2	3.40E-04	253666	98B	
	*CG32636*	2.5	6.20E-04	3.9	4.60E-04	NA	NA	
	*CG8026*	2	2.50E-04	3.8	4.90E-04	238705	45B	
	*Hdc*	2.7	1.80E-03	3.6	7.20E-04	−119479	46F	
	*CG32715*	2.7	4.60E-07	3.1	6.80E-03	NA	NA	
	*CG12990*	7.4	2.00E-07	2.9	2.10E-03	−249793	16B	
	*CG5204*	2.6	1.30E-03	2.6	6.80E-03	4937	34A	1
	*CG5376*	4	1.30E-03	2.2	9.60E-03	2152	94B	1
	*Rbp1*	−2.2	8.40E-03	-2	3.50E-03	−388811	86C	1

1 While no staining of HSF was noted on polytene chromosomes at 87B, the maximum staining intensity of 5.0 was observed at 87C and an intensity of 3.0 at 87A, the two loci traditionally documented to be the sites of the *Hsp70* genes.

(A) Genes induced by at least 8-fold in either Kc cells or 3^rd^ instar larvae. B) Genes exhibiting a 2- to 8-fold change in expression in both Kc cells and 3^rd^ instar larvae.

Because the transcriptional profile of larvae greatly differed from cells, we investigated whether genes responsive to heat shock in larvae were any more likely to be associated with HSF. The result was similar to cells; the mean distance from the nearest HSF binding site to the TSS was greater than 88 kb and the median distance was nearly 35 kb.

**Figure 10:**
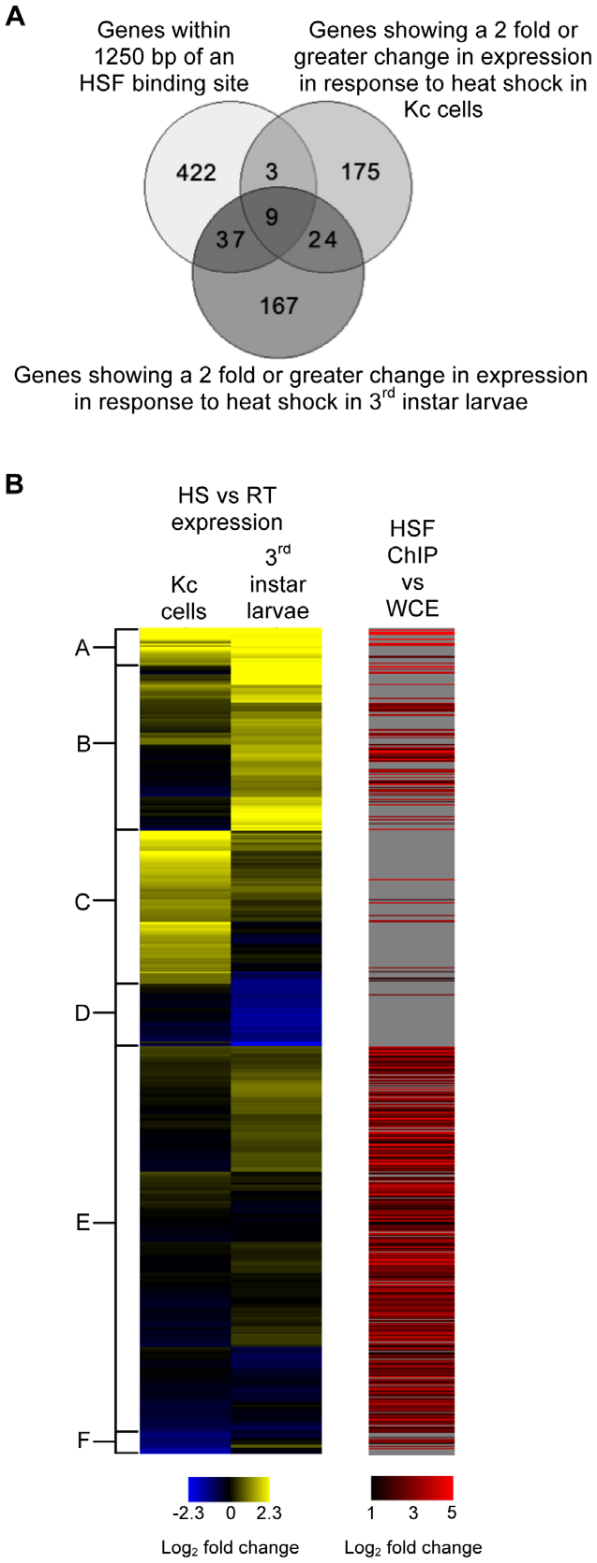
Many genes associated with HSF binding are not induced by HS in Kc cells or 3^rd^ instar larvae. (A) Venn diagram of the overlap between genes associated with an HSF-binding site and genes regulated by heat shock. (B) Hierarchical cluster of the HS transcriptional profile of all genes associated with an HSF-binding site identified in our ChIP-chip analysis and/or transcriptionally regulated by HS in cells and/or larvae (left). Sub-clusters highlight genes with similar expression profiles. For genes associated with an HSF-binding site, the log_2_ fold enrichment of the associated binding site is indicated in the aligned heat map (right). Where there is no associated binding site, the fold enrichment is displayed as grey to indicate no data is available.

To further investigate possible HSF association with genes regulated during heat shock we compared the lists of genes exhibiting a 2-fold or greater change in expression in response to heat shock in either cells or larvae to the 471 genes that either contained an HSF binding site within their coding region or were located 1250 bp downstream of the peak of an HSF bound region (Figure 10A). Only nine genes were in common to all three lists: *Hsp22*, *Hsp26*, *Hsp27*, *Hsp67Bb*, *Hsp68*, *Hsp70Ab*, *DnaJ-1*, *stv* and *CG32636*. Not surprisingly, most of these genes are well-known heat-inducible genes. *Starvin* (*stv*), although not a classical HSP, is induced in response to several stresses including oxidative and ER stress, aging, starvation and HS [27], [45], [46], [47], [48] and encodes a BAG-domain protein and is thought to be a Hsp70-family co-chaperone [49]. In addition to these nine genes, a total of 40 differentially regulated genes are located within 1250 bp of the peak of an HSF bound region. Thirty-nine of these are up regulated in response to HS in either cells or larvae but not both and one gene is down regulated in cells (Figure 10 and Table 3).

**Table 3:** Genes associated with HSF that are regulated by HS in either Kc cells, 3^rd^ instar larvae or both (see Figure 10).

	HS-regulated gene					HSF binding site		
	gene name	HS vs RT fold change (cells)	FDR corrected p-value (cells)	HS vs RT fold change (larvae)	FDR corrected p-value (larvae)	position of closest HSF binding site relative to TSS (bp)	HSF ChIP vs WCE fold change	HSF ChIP vs WCE segment min. P[Xbar]
Bound by HSF, induced in Kc cells and in 3rd instar larvae	*DnaJ-1*	5.3	3.25E-08	42	2.11E-05	219	27	1.18E-07
	*Hsp22*	14.1	3.70E-09	227	3.29E-08	−249	58	1.91E-04
	*Hsp26*	12.2	1.43E-09	28.5	1.12E-04	−305	75	8.76E-08
	*Hsp27*	8.8	7.56E-07	7.5	1.78E-04	−352	70	6.69E-08
	*Hsp67Bb*	2.1	7.53E-04	14.1	1.58E-04	838	58	1.91E-04
	*Hsp68*	24.7	2.24E-10	106.8	4.23E-08	−74	75	6.82E-08
	*Hsp70Ab*	31.7	3.30E-09	169.9	8.76E-10	−732	73	1.95E-10
	*stv*	3.9	3.73E-08	29.4	7.13E-10	−421	36	5.50E-07
	CG32636*	2.5	6.24E-04	3.9	4.60E-04	315	12	1.15E-06
Bound by HSF, induced in 3rd instar larvae	*CG10924*	1.5	1.70E-01	2.5	1.12E-04	2156	17	1.44E-10
	*CG10973*	1.2	2.51E-02	3.8	8.58E-03	97	20	2.30E-07
	*CG11033*	1.1	2.37E-01	2.1	9.46E-04	−740	28	1.46E-04
	*CG11035*	−1.6	1.69E-02	5.3	9.67E-08	−620	31	4.50E-11
	*CG13472*	−1.1	1.55E-01	3	3.24E-04	115	12	9.33E-06
	*CG1416*	−1.1	1.32E-01	2.7	8.87E-04	410	7	1.35E-08
	*CG1553*	−1.1	2.19E-01	4.4	8.86E-08	144	19	9.64E-11
	*CG1863*	−1.1	5.19E-01	5.6	7.49E-04	909	16	1.17E-10
	*CG32103*	−1	9.83E-01	2.3	3.60E-03	−472	5	7.73E-05
	*CG3226*	1.1	2.38E-01	3	6.09E-05	−125	33	1.25E-07
	*CG5010*	1.1	6.17E-01	3.4	1.27E-03	−376	26	1.70E-07
	*CG5290*	−1	8.58E-01	15.3	6.80E-08	81	34	4.31E-07
	*CG5953*	−1	8.89E-01	2.9	8.41E-04	2125	6	1.96E-06
	*CG6191*	−1.3	1.47E-03	2.2	1.93E-03	1430	16	8.42E-11
	*CG6511*	−1.1	2.96E-02	7.8	1.34E-06	36	28	2.24E-07
	*CG7945*	1.1	7.28E-01	5.2	1.13E-05	345	17	9.31E-06
	*CG9153*	−1.1	3.34E-01	2.6	6.02E-04	260	26	1.93E-07
	*Droj2*	−1.1	1.56E-01	2.7	2.57E-04	823	36	2.57E-11
	*GstD10*	1.6	5.04E-02	2.3	2.40E-05	−447	7	6.25E-07
	*Hop*	−1.1	2.39E-01	2.9	1.22E-04	108	73	3.23E-09
	*Hsc70-3*	1.1	2.39E-01	2.9	4.24E-04	1721	11	5.00E-05
	*Hsc70-4*	−1.1	1.03E-01	2.1	3.16E-06	−123	65	3.64E-12
	*Hsc70-5*	−1.1	8.90E-02	3.4	2.17E-05	236	18	1.05E-10
	*Hsc70Cb*	−1.1	2.35E-01	2.7	1.47E-04	195	30	1.31E-07
	*Hsp83*	1.3	2.40E-03	15.1	2.69E-06	5	71	7.58E-08
	*PEK*	−1.2	1.14E-01	3.7	3.83E-04	1048	6	5.22E-05
	*Pdk*	−1.1	5.87E-01	2.6	6.91E-05	4359	10	4.05E-10
	*Sir2*	−1	9.09E-01	2.8	9.63E-03	−87	76	3.73E-11
	*Taf7*	1.1	1.44E-01	7.3	6.86E-05	353	18	7.06E-11
	*Tom34*	−1.1	3.90E-01	6.5	9.74E-05	263	31	4.50E-11
	*cn*	−1.4	3.13E-01	2.2	4.62E-04	2174	17	1.25E-09
	*l(1)G0469*	1.8	3.85E-03	3.8	6.01E-03	2448	21	5.05E-07
	*mbf1*	1.2	5.24E-02	2.3	1.96E-04	−67	28	1.20E-06
	*pall*	1.1	2.57E-01	3.3	4.90E-04	497	5	6.21E-05
	*sra*	1	9.32E-01	3.3	1.99E-03	25	18	1.86E-10
	*tra*	−1.1	2.42E-01	3.2	4.37E-03	248	5	1.10E-04
	*ttk*	1.7	7.65E-03	2.3	3.18E-03	2452	13	7.08E-05
Bound by HSF, induced in Kc cells	*CG10077*	2.7	4.10E-03	7.8	1.38E-02	−104	22	4.39E-07
	*CG6770*	3	1.25E-03	1.7	2.03E-01	−390	35	2.43E-11
Bound by HSF, repressed in Kc cells	*GstD2*	−2.2	4.66E-03	−1.3	4.43E-01	−1110	6	1.23E-08

Because the FDR corrected p-value cutoff we applied in the identification of transcriptionally regulated genes of 0.01 is relatively stringent, it is possible that other genes associated with HSF-binding sites were transcriptionally regulated during HS but were not identified in our expression analysis. To investigate this possibility we generated a cluster of the expression profiles of all genes that were associated with an HSF binding site and/or were identified in our expression analysis of HS cells and larvae and aligned a heat map depicting the relative enrichment of the nearest HSF bound segment identified in our ChIP-chip analysis (where applicable; if no HSF-binding was located either within the coding region of the gene or <1250 bp upstream of the gene's TSS then the corresponding value in the heat map is grey indicating no data is available) (Figure 10B). This analysis revealed several things in support of our initial observations: First the majority of HS responsive genes exhibit an increase in transcript levels and are responsive in only one system even at relatively weak fold changes (Figure 10B clusters B and C). Second, the majority of HS-responsive genes, especially in cells, are not associated with HSF binding (Figure 10B clusters B, C, D and F). Finally, although there are a few HSF-associated genes that appear to undergo a small induction (less than 2-fold) in response to HS in larvae, the majority of HSF-associated genes are not transcriptionally responsive to HS in either cells or larvae (Figure 10 cluster E).

Of the 11 HSF-bound and HS-induced genes identified in Kc cells (Table 3) all are bound by HSF within 1250 bp of their annotated TSS (Table S3). Given the enrichment of BEAF motifs in HSF-bound promoter segments (Figure 9) we were interested in determining if there is any correlation between the presence of a BEAF motif and the likelihood of that gene to be expressed following HS. BEAF-binding sites are enriched in 5′ UTRs and in the first 200 bp upstream of gene TSS [40], [41] so we wanted to take the analysis one step further and examine the relationship between the presence of a BEAF motif and the induction of the associated gene during HS. Of the 11 HSF-bound promoters associated with HS-induced genes in Kc cells, only 3 or 27% were found to contain a BEAF motif (p-value<5×10^−4^) (Table 4). Conversely, of the 104 HSF-binding sites located exclusively in promoters (ie. within 1250 bp) of genes that did not show a transcriptional change following HS in Kc cells, 69 or 66% were found to contain at least one BEAF motif. Given this difference in the distribution of BEAF motifs between induced and non-induced HSF-bound promoters, we were interested in determining if BEAF is preferentially bound to promoters of non-induced genes under non-HS condition prior to exposure to HS. To examine this possibility we compared HSF-bound chromatin segments to chromatin segments bound by BEAF in Kc167 cells under non-HS conditions [40] (data available in GEO under GSE15661) and found that the proportion of HS-induced and non-induced HSF-bound promoters that were also bound by BEAF to be similar to the proportion containing at least one BEAF motif (Table 4) and that there is an enrichment for BEAF binding sites in non HS-induced HSF-bound gene promoters (Chi squared test; p-value  = 0.0198). Next we sought to determine if any other *Drosophila* insulators also co-localize with HSF binding sites or if this observation is specific for BEAF. We compared our HSF bound sites to the binding sites for two other insulators (dCTCF and Su(HW)) and an insulator associated protein, CP190, for which ChIP-chip data is available in Kc cells [40]. We did not see a significant enrichment for dCTCF or Su(HW) binding sites at HSF sites (data not shown) but we did see a large overlap with CP190 sites which is expected since CP190 does not bind DNA directly but does bind to insulators including BEAF [40]. This suggests that the enrichment of BEAF sites at HSF bound promoters is specific to BEAF and not a general feature of all insulators.

**Table 4:** Occurrence of BEAF motifs and binding sites in HSF-bound promoters.

	Promoters of genes induced during hs in Kc cells	Promoters of genes not induced during hs in Kc cells
Number of promoters with at least one BEAF motif (p-value<5×10-4)	3	69
Number of promoters bound by BEAF under non-hs conditions (Bushey et al., 2009)	3	66
Number of promoters bound by BEAF under non-hs conditions with at least one BEAF motif	2	48
Total number of promoters	11	104

Because there were a large number of genes whose transcripts changed during HS but did not appear to be near an HSF binding site, we investigated whether the transcriptional changes were dependent on HSF. The transcriptional response to HS was measured in *Hsf^4^* mutant 3^rd^ instar larvae using the same approach described above (data available in GEO under GSE22332). These larvae have a temperature sensitive mutation in the HSF DNA binding domain which prevent HSF from binding to HSEs and inducing HSP gene transcription at non-permissive (i.e. heat shock) temperatures [14]. Only 8 genes were up-regulated in the *Hsf^4^* larvae and the degree of induction was generally far less than what was seen in the wild type larvae (Table S3). Thus, it would appear that the vast majority of transcript levels that change during heat shock are dependent on having functional HSF.

### Heat shock represses the ecdysone-response

**Figure 11:**
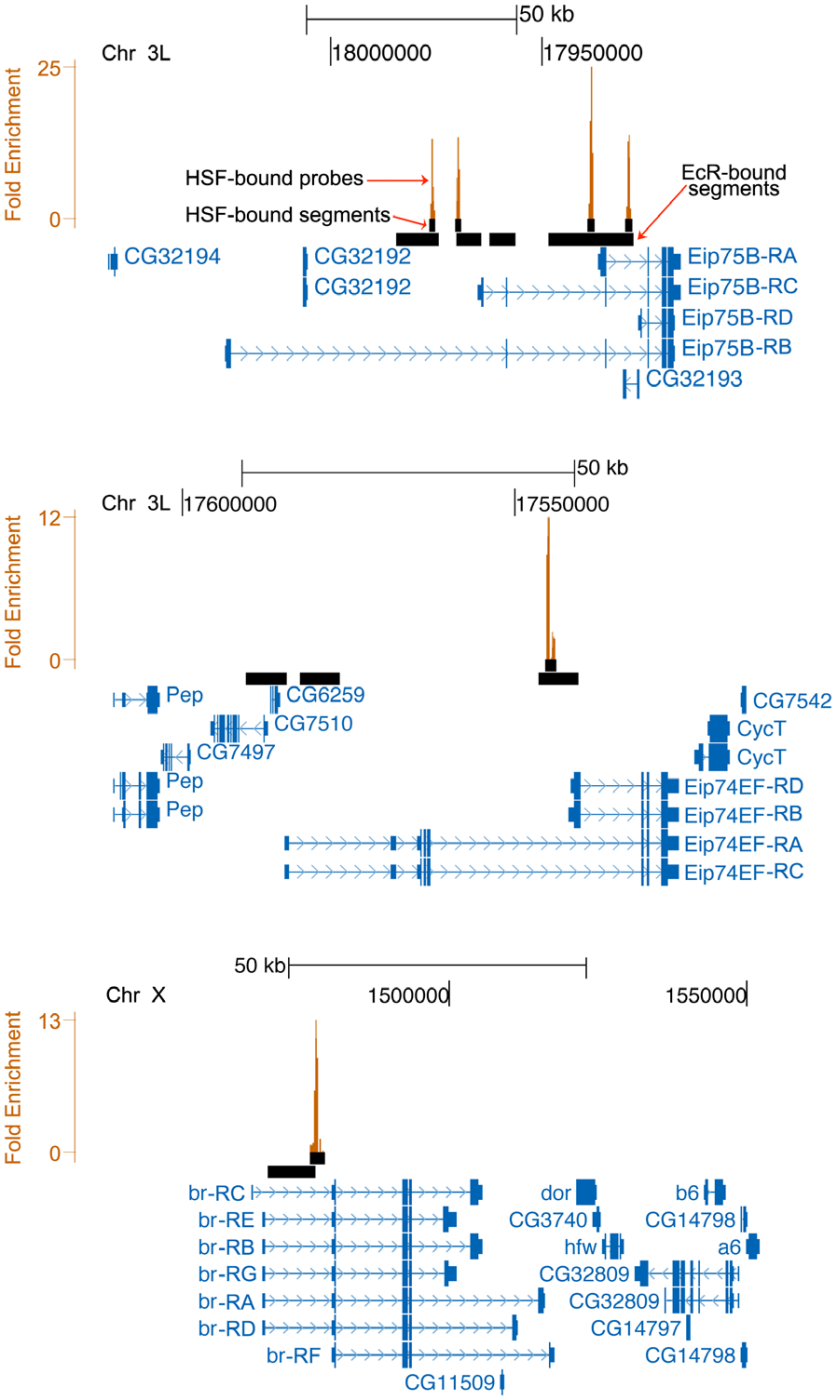
HSF binds to introns in the three major ecdysone-inducible genes, *Eip75B*, *Eip74EF*, and *br*. We have identified six HSF-binding sites within the bodies of these three genes. Each site is located within an intron and overlaps with a binding sites for the ecdysone receptor complex (EcR-C) [51]. Five out the six sites are also located within 5 kb of the transcription start site of one or more isoforms and the sixth site is still only 10 kb away from a transcription start site. Each orange bar represents a probe from the tiling array that is part of an HSF bound segment and its height indicates its fold enrichment relative to WCE. As with Figure 4, this image was generated using the UCSC Genome Browser to illustrate the chromosome region represented in bp (according to release 4.2 of the *Drosophila* genome) as indicated at the top and the legend is the same as that in Figure 4.

Since HSF binds a large number of introns of genes that do not appear to be transcriptionally induced during HS, we decided to examine a subset of HSF-bound introns more closely. The functional enrichment analysis presented in the previous section (Figure 8), indicates that intron-bound genes are strongly enriched in functions related to both the regulation of transcription and to developmental processes. We had noted in a previous study that HSF binds to two well examined chromosomal loci, 74EF and 75B, the sites of two of the early ecdysone inducible transcription factor genes, *Eip74EF* and *Eip75B* [32]. These two genes together with *br*, are major components of the transcription hierarchy that are involved in the developmental response to ecdysone by regulating the transcription of a large set of secondary ecdysone-response genes (for review see [50]). All three of these genes are very large in size spanning from 59 to over 100 kb and each encodes multiple isoforms with at least two distinct transcription start sites (for review see [50]). Within these three genes, we have found a total of six HSF-binding sites, four within *Eip75B*, one within *Eip74EF* and one within *br* (Figure 11). Interestingly, all but one of these intronic HSF-binding sites are located less than 5 kb upstream of 5′ end of one or more isoforms. Given that introns in these genes that are bound by HSF are rather large (for example the first intron of *Eip75B* in which HSF occupies two sites, is over 60 kb in length and the intron bound by HSF in *Eip74EF* is over 20 kb) this distribution of HSF-binding sites appears to exhibit a strong bias for the extreme 3′ end of the introns close to the 5′ ends of alternate isoforms. In addition, all five HSF binding sites overlap with binding sites identified in Kc167 cells for the ecdysone receptor complex (EcR-C) [51], the nuclear receptor complex responsible for the stage and tissue specific activation of *Eip75B*, *Eip74EF*, and *br* [52], [53].

**Figure 12:**
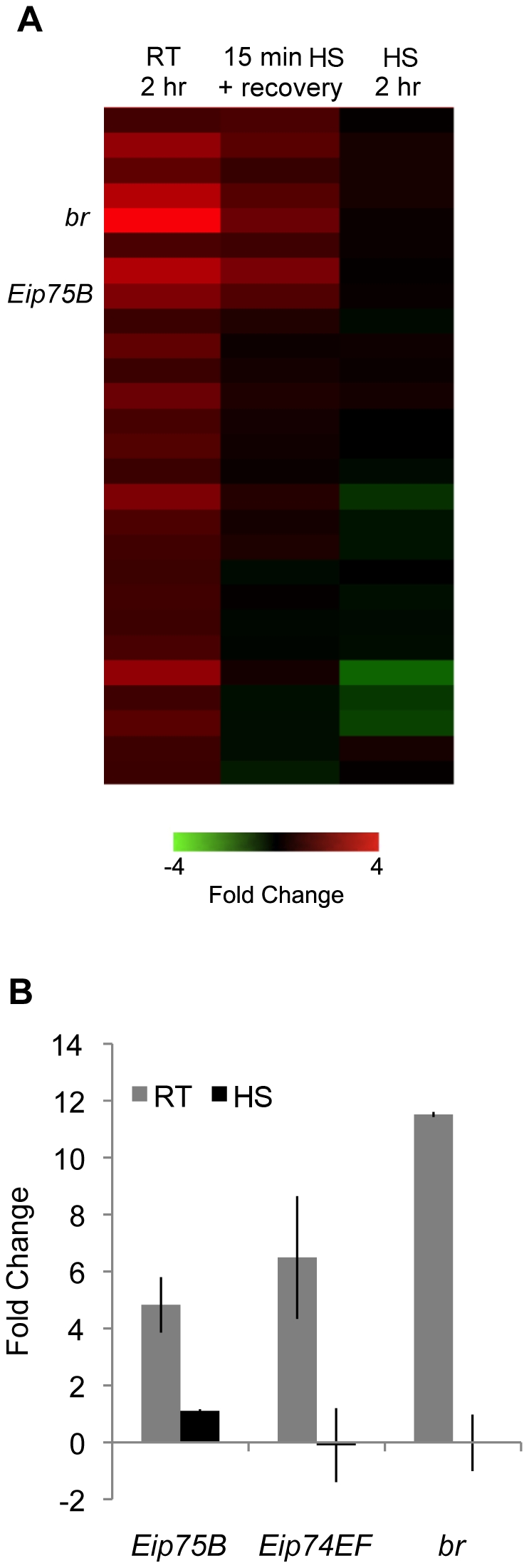
Heat shock prevents transcription of ecdysone-regulated genes in Kc cells. (A) Microarray analysis identifies 27 genes that are induced at least 1.5 fold in Kc cells by a 2 hr 0.5 µM ecdysone treatment under non-HS conditions (left most column). The number of genes that respond transcriptionally to ecdysone is greatly reduced when ecdysone is administered immediately following a brief, 15 min, HS. Even those genes that are still induced by ecdysone are induced to a lesser extent (middle column). When subjected to HS, however, the same genes that were induced by ecdysone under non-HS conditions, no longer respond transcriptionally to the same ecdysone treatment (ie. 2 hr 0.5 µM ecdysone) indicting that HS can repress ecdysone-inducible gene transcription. (B) Quantitative RT-PCR verification of the microarray results for the major ecdysone-inducible genes *Eip75B*, *Eip74EF*, and *br* yields the same conclusion. All three genes are induced when exposed to 2 hr 0.5 µM ecdysone treatment at RT (grey bars) but not during HS (black bars). Error bars represent standard error.

It has been previously shown that the developmentally regulated chromosomal puffs like the ecdysone induced puffs regress during HS [32], [54]. Given that HSF-binding coincides with puff regression at these loci, we were interested in determining if HS would affect transcription of ecdysone-response genes so we examined the global transcriptional response of Kc cells to a 2 hr ecdysone treatment followed by both transient (15 min HS followed by 2 hr ecdysone treatment at RT) and sustained exposure to HS (HS for the entire duration of the 2 hr ecdysone treatment) (Figure 12A; data available in GEO under GSE23824). Our results support the previous observation that HS results in the repression of global gene expression including genes that are directly associated with HSF-binding sites such as *Eip75B*, *Eip74EF*, and *br* [32]. The magnitude of repression is related to the duration of the stress; prolonged exposure to HS inhibits gene expression to a much greater degree than exposure to brief and transient HS treatment and ecdysone-induced gene transcription starts to recover to normal levels following removal of the stress with the primary ecdysone-response genes being the first to return to normal levels (Figure 12A). Given that HSF binding within the body of these genes is coincident with their repression it is possible that HSF may have a direct role in the repression of these genes. It is not clear, however, from the location of the HSF binding sites if HSF may be interfering with transcription initiation form alternate promoters in the vicinity of its binding site or if may be interfering with transcription from upstream promoters by an obstruction to transcription elongation. The mammalian HSF homolog, HSF1, is known to cause repression of prointerleukin 1β and Tumor Necrosis Factor α [33], [55], although in the present case it is not clear if the mode of action would be the same.

## Discussion

HSF in metazoans is activated upon stress to trimerize and bind HSEs that are found in the promoters of HSP genes. HSF binding leads to the release of stalled RNA polymerase II as well as the recruitment of new polymerase complexes [56]. It has long been known that HSF binds to many other parts of the genome in addition to the well known HS gene loci [32].

In this study we attempted to determine all of the HSF binding sites in *Drosophila melanogaster* using ChIP-chip methodology on *Drosophila* genomic tiling arrays. In total we identified 434 HSF bound chromatin segments in heat shocked Kc167 cells with the transcription start site (TSS) of 270 genes mapping to within 1250 bp of an HSF binding site. A comparison of our binding data to an earlier study that utilized heat shocked *Drosophila* embryos and cDNA arrays for the ChIP-chip shows that approximately 33% of the HSF-bound genes in their study (i.e. 62 out of 188) correlate with our binding sites (for a comparison of the overlapping sites see Table S2) [29]. The binding sites we identified correlate quite well with those identified by another group that used ChIP-seq to uncover HSF binding sites in S2 cells [31]. 263 of the 442 high confidence HSF binding sites found in that study coincided with HSF binding segments we found (Table S2). If we look at our top 100 HSF bound segments, 90 were also identified as HSF binding sites by this group. Differences between the studies might be due to several factors- differences in the cell types, antibodies, array platform, and ChIP or sequence identification methods that were used as well as differences in the analyses that were performed. Others have noted variation in transcription factor binding sites (i.e. for NFκB) in ChIP-seq experiments for different biological individuals even though the same cell type and identical experimental procedures were utilized [57].

We attempted to correlate the HSF binding events with changes in gene transcription using standard expression microarray analysis of heat shocked Kc cells and *Drosophila* 3^rd^ instar larvae. These experiments revealed a number of surprising results. First, the transcript profiles of heat shocked Kc cells and 3^rd^ instar larvae were quite different (Figure 10). For example, even though each system resulted in more than 200 differentially expressed transcripts, only 33 or about 8% of these transcripts were in common to both systems and of these genes, only 9 had HSF bound segments within 1250 bp of a TSS. Second, only 49 (or about 11%) of the HSF binding sites were found to be within 1250 bp of a TSS for a differentially expressed gene in either system. This suggests that the majority of differentially expressed genes are either being regulated by HSF from a distance more than 1250 bp away or that the differential levels in this class of transcripts is being regulated by a different mechanism. We ruled out the possibility of these transcripts being regulated by an HSF-dependent newly synthesized transcription factor by repeating the experiment in the presence of cycloheximide which did not significantly alter the list of differentially expressed genes induced by heat. We also determined that the vast majority of transcript changes that occur during HS are dependent on having functional HSF since larvae that have a mutated HSF gene (*Hsf^4^* larvae) show very few changes in transcript levels. For these few genes, differential levels of these transcripts might be regulated post-transcriptionally, a phenomenon reported for certain HSP genes in *Hsf^4^* flies [58]. How HSF might be regulating the other transcripts is still not clear and we cannot rule out that HSF may be interacting with other transcription/chromatin factors without binding to a nearby HSE. HSF could also be working at distances greater than 1250 bp since in three dimensional space, the binding of HSF may in fact be much closer to a TSS. We also cannot rule out that the ChIP-chip approach we used did not uncover all of the HSF binding sites in the genome.

We were also interested in determining if any other transcription factor(s)/DNA binding protein(s) were associated with these HSF sites. We did find that a number of HSF bound promoters (and not introns) also contained binding sites for the BEAF transcription factor (known to be important for insulating enhancers) and this finding is consistent with a recent report that BEAF-binding sites tend to be associated with the 5′ UTRs and regions immediately upstream of the transcription start site of genes [40], [41]. Interestingly, we found an enrichment for BEAF binding under non-HS conditions [40] to promoters of genes that are bound by HSF but that are not induced during HS. Since a proportion of the BEAF-binding sites identified in Kc cells is cell type specific [40], [41], it is possible that BEAF may have a role preventing the induction of genes near select HSF-binding sites during HS in a cell specific manner.

A recent paper by Guertin and Lis [31] investigated the distribution of chromatin modifications and certain chromatin proteins at HSEs prior to the binding of HSF in *Drosophila*. Overall, they observed a correlation of histone acetylation, H3K4 trimethylation, RNA polymerase II and coactivators such as GAGA factor with HSEs that ultimately are bound by HSF after heat shock compared to HSEs that are not bound by HSF. These chromatin modifications and proteins are hallmarks of transcriptionally active chromatin and the authors argue that the modifications are requirements for HSF to bind to HSEs prior to transcriptional induction as opposed to a consequence of transcription [31]. These authors also noted a large number of HSF binding sites that were also bound by BEAF prior to HS with a higher occurrence of the overlap taking place at promoters than within genes. Moreover, for the few HSF associated genes whose transcription were examined after HS, BEAF binding was more enriched at non-induced compared to induced genes [31].

What is the function of HSF binding to so many different places in the genome if it is not to regulate the heat shock genes during stress? There is the possibility that there are some genes that are being transcriptionally induced by HSF during HS in other developmental stages and/or tissue types. The different transcriptional response to heat by Kc cells and 3^rd^ instar larvae lends support to this hypothesis. That is, the transcriptional response to an active transcription factor is likely dependent on the cellular/nuclear environment and/or chromatin state that exists in a given cell type. As discussed above, this could include the possibility that HSF might be acting as a specific transcriptional repressor of certain developmentally regulated genes whose puffs on polytene chromosome regress during heat shock [32]. A similar difference between binding events and transcriptional responses has also been seen for the Ecdysone receptor/Ultraspiracle nuclear hormone complex binding sites and the transcription profiles seen in Kc167 cells and the during *Drosophila* metamorphosis [51].

Another possibility is that HSEs appear in the genome with a certain frequency and have no biological consequence. It has been suggested that there are a large number of cis-regulatory modules (CRMs) in the *Drosophila* genome that fall into this category [59]. Natural selection would preserve those CRMs that are critical to transcriptional regulation but an organism could tolerate CRMs that had weak affinity for a given transcription factor that did not interfere with transcriptional regulation [59], [60]. The existence of large numbers of transcription factor binding sites that have no apparent biological activity would appear to be a property of all eukaryotic organisms [61].

Yet another possibility is that HSF has functions during non-HS conditions and that the ChIP-chip analysis is revealing many of those gene targets. Clearly HSF deficient *Drosophila* show developmental arrest (i.e. at 1^st^ and 2^nd^ instar) as well as defects in oogenesis under non-HS conditions [14]. In species such as *Drosophila* that have a single HSF, HSF may be performing numerous roles under both HS and non-HS conditions. It is also possible that during *Drosophila* development that other forms of stress are occurring that induce HSF transiently. As animals evolved, gene duplication and divergence resulted in multiple HSFs that distributed some of the important functions to specific and/or multiple HSFs. As previously mentioned, mice lacking HSF1 display growth retardation and female infertility due to defective oocytes [15], [16]. *Hsf1 ^−/−^* oocytes exhibit a delay and blockage of meiotic maturation and this defect at least in part can be related to a decrease in *Hsp90α* transcript levels and Hsp90 protein activity [62]. *Hsf2^−/−^* mice show embryonic brain defects with the defect in cerebral cortex formation being attributed to the reduced expression of an HSF2 regulated gene, *p35* [63]. Both HSF1 and HSF2 have been shown to play roles in sperm development in mice with *Hsf2^−/−^* mice showing a more severe defect resulting in a reduced testis size and the disruption of spermatogenesis characterized by degenerating cells, the absence of differentiating spermatids and spermatocytes, vacuolization of the tubules and reduced sperm count [20], [21]. A mouse HSF2 ChIP-chip study performed with testis found numerous promoters that bound HSF2 including almost 1/3^rd^ of the 105 genes known to exist on the Y chromosome [30]. HSF2 was found to bind and regulate multi-copy genes in the male-specific region of the Y chromosome (MSYq) and HSF2 deficient mice had similar increases in sperm head defects as those with MSYq deletion mutations [30].

It is interesting that the DAVID analysis of the HSF binding sites in *Drosophila* showed enrichment for a number of developmental processes as well as gamete formation including oogenesis (Figure 8). Also revealing was that this enrichment was only seen for the HSF binding sites found in the introns of genes whereas the analysis of HSF binding sites in promoters and introns and in the genes that showed transcriptional changes showed enrichment in GO categories such as response to stress and transferase activity.

## Methods

### Cell culture and heat-shock treatments

*Drosophila* Kc167 cells [34], obtained from the *Drosophila* Genomic Resource Center (Indiana University, Bloomington) were grown to confluence in Schneider's media (Invitrogen) supplemented with 5% heat-inactivated FBS (Sigma) and 20 µg/ml gentamicin (Sigma) in tissue culture flasks at 22°C. Prior to heat-treatment, cells were transferred to Erlenmeyer flasks and aerated for 4 hrs at 22°C by gentle shaking (∼180 rpm). Following aeration, half of the cells were heat-shocked by submersing the flask in a 36.5°C circulating water bath (Neslab) for 30 minutes. The remaining cells were maintained as room temperature controls. For the cycloheximide experiments, 118 µM cycloheximide was added 10 minutes prior to initiating the heat shock treatment [64] which was otherwise carried out as stated above. For the ecdysone experiments, cells were treated with 0.5 µM 20-hydroxyecdysone (Sigma) for 2 hrs at room temperature either with or without a 15 minute pre-treatment with heat shock (36.5°C) or for 2 hrs at heat shock temperatures (36.5°C).

### ChIP

Cell cross-linking, lysis and chromatin shearing were all performed as reported in [65]. Dynabead protein G magnetic bead (Invitrogen Cat. No. 100.03D) preparation, immunoprecipitation, immunocomplex elution, cross-link reversal, and DNA precipitation were all performed according to [66]. Rabbit polyclonal anti-HSF serum 943 [32] was used at a dilution of 1:7500.

### End-point and qPCR

Standard PCR was performed on DNA from HS and RT, anti-HSF and mock ChIPs, and WCE with primers designed against the *Hsp26* promoter and on DNA from unamplified and amplified HS anti-HSF and mock ChIPs with primers designed against regions upstream of the following genes: *Hsrω, Hsp70Ab, Hsp83, CG11267, stv, CG5290, Tom34, DnaJ-1, CG10077, Taf7,* and *GstD2*. Cycling conditions used are as follows: 95°C for 3′ followed by 35 cycles of 95°C for 30”, 58°C for 45” and 72°C for 30”. Quantitative PCR was performed on the same DNA samples with the same primers plus additional primers designed to amplify the region of chromosomal DNA 1200 bp downstream of the *Hsp26* promoter. Our qPCR reactions were performed with Brilliant SYBR Green (Stratagene) in a MX4000 lightcycler (Stratgene) under the same conditions as above (only for 40 cycles instead of 35). See Table S4 for the sequences of the primers used.

### Ligated-Mediated PCR

The immunoprecipitated sheared chromatin was repaired as described in [66]. Linker DNA used in ligation-mediated PCR (LM-PCR) was prepared according to [29] as was ligation of the repaired DNA to the linker and PCR amplification of the ligated chromatin.

### Indirect labeling of amplified chromatin and hybridization to genome-tiling arrays

A second round of PCR similar to that performed for LM-PCR was used to incorporate amino-allyl modified nucleotides into the amplified material. Following amplification, DNA clean-up, fluorescent dye conjugation and probe clean-up and precipitation was performed as described on the Canadian *Drosophila* Microarray Centre web site (www.flyarrays.com). Labeled DNA was mixed with control nucleic acids (750 ng salmon sperm DNA, 40 µg yeast tRNA, 10 µG human cot-1 DNA) and then added to hybridization buffer (50 mM Na-MES pH 6.9, 500 mM NaCl, 6 mM EDTA, 0.5% ultrapure sarcosine, 30% ultrapure formamide) heated to 95°C for 3 min and then incubated at 40°C for 15 min. Labeled DNA was hybridized to Agilent genome-tiling arrays containing approximately 475,000 60mer probes (i.e a probe every 233 nucleotides) according to manufacturer's directions. After 20 hours of hybridization, the slides were washed for 5 min with 6x SSPE, 0.005% ultrapure N-lauroylsarcosine, again for 5 min, 0.6x SSPE, dipped in acetonitrile and washed for 30 s in Agilent's Wash III. Dried slides were scanned with Agilent's microarray scanner and the resulting images were quantified with Agilent's Feature Extraction software.

### HSF binding site identification

Data from Feature Extraction was normalized with Agilent's ChIP Analytics software. Blank subtraction normalization, inter-array median normalization and intra-array (dye-bias) median normalization were all applied. Probes were mapped to release 4.2 of the *Drosophila* genome. The Whitehead Error Model v1.0 and Whitehead Per-Array Neighborhood Model v1.0 were used with the default settings for error modeling and for peak detection/evaluation, respectively with a false discovery rate of 11%.

### PWM matching

We used the pattern matching program Patser [38] to find matches to four PWMs from TRANSFAC representing canonical (M00165) and non-canonical (M00163, M00164, M00166) HSEs in the sequence flanking each identified bound peak (+/−2500 bp). For each matrix, we counted the number of matches with p-values below 1×10^−4^ and 1×10^−5^ in 50 bp windows relative to the segment peak and generated a frequency histogram. To determine if there is a significant difference in the number of matches in the region immediately surrounding the peak (−500 bp to +500 bp) relative to the local background (−1000 bp to −550 bp and +550 bp to +1000 bp) we performed a X^2^ test. We repeated these steps to identify matches to all other unique PWMs from TRANSFAC and from the *Drosophila* DNase I Footprint Database this time counting matches to each matrix in HSF-bound promoters and HSF-bound introns with Bonferroni corrected p-values <5.6×10^−2^. To control for base composition bias of the test sequence, for any matrix exhibiting an enrichment of binding sites in the peak region relative to the local background comparable to the enrichment seen for the canonical HSE, we repeated the test with a scrambled version of the matrix and then threw out any matrix still showing enrichment. For any remaining matrices, we performed a X^2^ test to determine if there is a significant difference in the number of sites matching the matrix in the HSF-bound promoters relative to HSF-bound introns.

### Larval heat shock treatment

Late third instar larvae (*dp cn bw* and *Hsf^4^ cn bw*) [14] were selected by the blue gut method as previously described [67] and transferred to 2 ml screw cap tubes containing a strip of moist blotting paper with no more than 20 larvae per tube. Larvae were allowed to acclimatize for 1 hour at RT with loose lids and then either submerged in a 36.5°C circulating water bath (Neslab) for 30 min or kept at RT (22°C) for the same amount of time. Following treatment, larvae were snap frozen in liquid nitrogen.

### RNA extraction cDNA synthesis, and labeling and hybridization to expression microarrays

Total RNA was extracted from both cells and larvae using TRIzol reagent (Invitrogen) according to the manufacture's protocol. Quality and quantity of RNA was verified by measuring the absorbance and the A260/A280 ratios were always above 1.8. cDNA synthesis, labeling and hybridization to *Drosophila* 385 k NimbleGen expression microarrays (Roche) was carried out as described in the manufacture's protocol with the exception that HS and RT samples were differentially labeled and hybridized to a single array. For each treatment, three independent biological replicates were performed. For the ecdysone plus heat shock experiments, a cDNA based microarray was used and the microarray experiments and analysis were carried out following the methods of Neal and co-workers [68].

### Expression microarray data extraction and analysis

Images acquired after scanning slides with GenePix 4000B microarray scanner (Molecular Devices) were quantified and RMA normalized with NimbleScan (Roche). ArrayStar (DNASTAR) was used to analyze the resulting data files and identify genes with an average fold change across all biological replicates of 2 fold or greater and FDR corrected p-values less than 0.01. Log-converted expression ratios were clustered in the microarray data analysis software MeV [69], [70] using the Manhattan Distance Metric and average linkage method.

### Functional Enrichment of Gene Lists

Functional enrichment analysis was performed using the Database for Annotation, Visualization, and Integrated Discovery Bioinformatics Resources (DAVID) [39], [71]. Lists of Flybase Gene Identifiers from genes that were differentially expressed during HS in either cells or larvae and from genes that were bound by HSF in either the promoter regions or intronic regions were input into the functional annotation clustering tool and functional annotation chart tool. For genes with HSF binding sites in their intronic regions, annotations were compared to the pool of annotations found for all genes with introns, whereas all other lists were compared to the entire genome. Highly related groups of enriched annotations were identified from each of the 4 gene lists and the corresponding p-values from the DAVID analysis for those annotation terms from each of the lists were compared to each other using a heat map.

## Supporting Information

Table S1 **HSF binding ratios of segments bound under hs and non-hs conditions.**
(XLS)

Table S2 **Complete list of sites and associated genes bound by HSF.**
(XLS)

Table S3 **Complete list of genes exhibiting a 2 fold or greater change in response to heat shock in either Kc cells, wild type (** ***dp*** **), and** ***Hsf*** **mutant (** ***Hsf^4^*** **) larvae.** The position of the nearest HSF binding site to each gene is also given.
(XLS)

Table S4 **Primer sequences used for gene specific PCR.**
(XLS)

## References

[1] Ritossa F (1962). A new puffing pattern induced by temperature shock and DNP in *Drosophila*.. Experientia.

[2] Parsell DA, Lindquist S (1993). The function of heat-shock proteins in stress tolerance: Degradation and reactivation of damaged proteins.. Annu Rev Genet.

[3] Young JC, Agashe VR, Siegers K, Hartl FU (2004). Pathways of chaperone-mediated protein folding in the cytosol.. Nat Rev Mol Cell Biol.

[4] Barral JM, Broadley SA, Schaffar G, Hartl FU (2004). Roles of molecular chaperones in protein misfolding diseases.. Semin Cell Dev Biol.

[5] Amin J, Ananthan J, Voellmy R (1988). Key features of heat shock regulatory elements.. Mol Cell Biol.

[6] Perisic O, Xiao H, Lis JT (1989). Stable binding of *Drosophila* heat shock factor to head-to-head and tail-to-tail repeats of a conserved 5 bp recognition unit.. Cell.

[7] Xiao H, Lis JT (1988). Germline transformation used to define key features of heat-shock response elements.. Science.

[8] Fernandes M, O'Brien T, Lis JT, Morimoto RI, Tissières A, Georgopoulos C (1994). Structure and regulation of heat shock gene promoters.. The biology of heat shock proteins and molecular chaperones.

[9] Wu C (1995). Heat shock transcription factors: Structure and regulation.. Annu Rev Cell Dev Biol.

[10] Ho JS-L, Westwood JT, Locke M, Noble EG (2002). Transcriptional regulation of the mammalian heat shock genes.. Exercise and stress response.

[11] Voellmy R (2004). Transcriptional regulation of the metazoan stress protein response.. Prog Nucleic Acid Res Mol Biol.

[12] Pirkkala L, Nykanen P, Sistonen L (2001). Roles of the heat shock transcription factors in regulation of the heat shock response and beyond.. FASEB J.

[13] Cotto JJ, Morimoto RI (1999). Stress-induced activation of the heat-shock response: Cell and molecular biology of heat-shock factors.. Biochem Soc Symp.

[14] Jedlicka P, Mortin MA, Wu C (1997). Multiple functions of *Drosophila* heat shock transcription factor in vivo.. EMBO J.

[15] Xiao X, Zuo X, Davis AA, McMillan DR, Curry BB (1999). HSF1 is required for extra-embryonic development, postnatal growth and protection during inflammatory responses in mice.. EMBO J.

[16] Christians E, Davis AA, Thomas SD, Benjamin IJ (2000). Maternal effect of hsf1 on reproductive success.. Nature.

[17] Fujimoto M, Izu H, Seki K, Fukuda K, Nishida T (2004). HSF4 is required for normal cell growth and differentiation during mouse lens development.. EMBO J.

[18] Takaki E, Fujimoto M, Sugahara K, Nakahari T, Yonemura S (2006). Maintenance of olfactory neurogenesis requires HSF1, a major heat shock transcription factor in mice.. J Biol Chem.

[19] Bu L, Jin Y, Shi Y, Chu R, Ban A (2002). Mutant DNA-binding domain of HSF4 is associated with autosomal dominant lamellar and marner cataract.. Nat Genet.

[20] Kallio M, Chang Y, Manuel M, Alastalo TP, Rallu M (2002). Brain abnormalities, defective meiotic chromosome synapsis and female subfertility in HSF2 null mice.. EMBO J.

[21] Wang G, Zhang J, Moskophidis D, Mivechi NF (2003). Targeted disruption of the heat shock transcription factor (hsf)-2 gene results in increased embryonic lethality, neuronal defects, and reduced spermatogenesis.. Genesis.

[22] Salmand PA, Jungas T, Fernandez M, Conter A, Christians ES (2008). Mouse heat-shock factor 1 (HSF1) is involved in testicular response to genotoxic stress induced by doxorubicin.. Biol Reprod.

[23] GuhaThakurta D, Palomar L, Stormo GD, Tedesco P, Johnson TE (2002). Identification of a novel cis-regulatory element involved in the heat shock response in caenorhabditis elegans using microarray gene expression and computational methods.. Genome Res.

[24] Murray JI, Whitfield ML, Trinklein ND, Myers RM, Brown PO (2004). Diverse and specific gene expression responses to stresses in cultured human cells.. Mol Biol Cell.

[25] Trinklein N, Murray J, Hartman S, Botstein D, Myers R (2004). The role of heat shock transcription factor 1 in the genome-wide regulation of the mammalian heat shock response.. Mol Biol Cell.

[26] Leemans R, Egger B, Loop T, Kammermeier L, He H (2000). Quantitative transcript imaging in normal and heat-shocked *Drosophila* embryos by using high-density oligonucleotide arrays.. Proc Natl Acad Sci U S A.

[27] Sorensen JG, Nielsen MM, Kruhoffer M, Justesen J, Loeschcke V (2005). Full genome gene expression analysis of the heat stress response in *Drosophila melanogaster*.. Cell Stress Chaperones.

[28] Hahn J-S, Hu Z, Thiele DJ, Iyer VR (2004). Genome-wide analysis of the biology of stress responses through heat shock transcription factor.. Mol Cell Biol.

[29] Birch-Machin I, Gao S, Huen D, McGirr R, White RA (2005). Genomic analysis of heat-shock factor targets in *Drosophila*.. Genome Biol.

[30] Akerfelt M, Henriksson E, Laiho A, Vihervaara A, Rautoma K (2008). Promoter chip-chip analysis in mouse testis reveals Y chromosome occupancy by HSF2.. Proc Natl Acad Sci U S A.

[31] Guertin MJ, Lis JT (2010). Chromatin landscape dictates HSF binding to target DNA elements.. PLoS Genet.

[32] Westwood JT, Clos J, Wu C (1991). Stress-induced oligomerization and chromosomal relocalization of heat-shock factor.. Nature.

[33] Cahill CM, Waterman WR, Xie Y, Auron PE, Calderwood SK (1996). Transcriptional repression of the prointerleukin 1beta gene by heat shock factor 1.. J Biol Chem.

[34] Echalier G, Ohanessian A (1970). In vitro culture of *Drosophila melanogaster* embryonic cells.. In vitro.

[35] Shopland LS, Lis JT (1996). HSF recruitment and loss at most *Drosophila* heat shock loci is coordinated and depends on proximal promoter sequences.. Chromosoma.

[36] Wu C, Clos J, Giorgi G, Haroun R, Kim S-J, Morimoto RI, Tissières A, Georgopoulos C (1994). Structure and regulation of heat shock transcription factor.. The biology of heat shock proteins and molecular chaperones.

[37] Tweedie S, Ashburner M, Falls K, Leyland P, McQuilton P (2009). Flybase: Enhancing *Drosophila* gene ontology annotations.. Nucleic Acids Res.

[38] Hertz GZ, Stormo GD (1999). Identifying DNA and protein patterns with statistically significant alignments of multiple sequences.. Bioinformatics.

[39] Dennis G, Sherman BT, Hosack DA, Yang J, Gao W (2003). DAVID: Database for annotation, visualization, and integrated discovery.. Genome Biol.

[40] Bushey AM, Ramos E, Corces VG (2009). Three subclasses of a *Drosophila* insulator show distinct and cell type-specific genomic distributions.. Genes Dev.

[41] Gurudatta BV, Corces VG (2009). Chromatin insulators: Lessons from the fly.. Briefings in Functional Genomics and Proteomics.

[42] Emberly E, Blattes R, Schuettengruber B, Hennion M, Jiang N (2008). BEAF regulates cell-cycle genes through the controlled deposition of H3K9 methylation marks into its conserved dual-core binding sites.. PLoS Biol.

[43] Greenleaf AL, Plagens U, Jamrich M, Bautz EK (1978). RNA polymerase b (or ii) in heat induced puffs of *Drosophila* polytene chromosomes.. Chromosoma.

[44] Jamrich M, Greenleaf AL, Bautz EK (1977). Localization of RNA polymerase in polytene chromosomes of *Drosophila melanogaster*.. Proc Natl Acad Sci U S A.

[45] Girardot F, Monnier V, Tricoire H (2004). Genome wide analysis of common and specific stress responses in adult *Drosophila melanogaster*.. BMC Genomics.

[46] Landis GN, Abdueva D, Skvortsov D, Yang J, Rabin BE (2004). Similar gene expression patterns characterize aging and oxidative stress in *Drosophila melanogaster*.. Proc Natl Acad Sci USA.

[47] Harbison ST, Chang S, Kamdar KP, Mackay TFC (2005). Quantitative genomics of starvation stress resistance in *Drosophila*.. Genome Biol.

[48] Zinke I, Schütz CS, Katzenberger JD, Bauer M, Pankratz MJ (2002). Nutrient control of gene expression in *Drosophila*: Microarray analysis of starvation and sugar-dependent response.. EMBO J.

[49] Coulson M, Robert S, Saint R (2005). *Drosophila* starvin encodes a tissue-specific bag-domain protein required for larval food uptake.. Genetics.

[50] Thummel CS (1990). Puffs and gene regulation—molecular insights into the *Drosophila* ecdysone regulatory hierarchy.. Bioessays.

[51] Gauhar Z, Sun LV, Hua S, Mason CE, Fuchs F (2009). Genomic mapping of binding regions for the ecdysone receptor protein complex.. Genome Res.

[52] Koelle MR, Talbot WS, Segraves WA, Bender MT, Cherbas P (1991). The *Drosophila* EcR gene encodes an ecdysone receptor, a new member of the steroid receptor superfamily.. Cell.

[53] Yao TP, Forman BM, Jiang Z, Cherbas L, Chen JD (1993). Functional ecdysone receptor is the product of EcR and ultraspiracle genes.. Nature.

[54] Ashburner M (1970). Patterns of puffing activity in the salivary gland chromosomes of *Drosophila*. V. Responses to environmental treatments.. Chromosoma.

[55] Xie Y (2002). NF-IL6 and HSF1 have mutually antagonistic effects on transcription in monocytic cells.. Biochem Biophys Res Commun.

[56] Rougvie AE, Lis JT (1988). The RNA polymerase ii molecule at the 5′ end of the uninduced hsp70 gene of *D. melanogaster* is transcriptionally engaged.. Cell.

[57] Kasowski M, Grubert F, Heffelfinger C, Hariharan M, Asabere A (2010). Variation in transcription factor binding among humans.. Science.

[58] Neal SJ, Karunanithi S, Best A, So AK-C, Tanguay RM (2006). Thermoprotection of synaptic transmission in a *Drosophila* heat shock factor mutant is accompanied by increased expression of Hsp83 and DnaJ-1.. Physiol Genomics.

[59] Macarthur S, Li X, Li J, Brown J, Chu H (2009). Developmental roles of 21 *Drosophila* transcription factors are determined by quantitative differences in binding to an overlapping set of thousands of genomic regions.. Genome Biol.

[60] Li XY, MacArthur S, Bourgon R, Nix D, Pollard DA (2008). Transcription factors bind thousands of active and inactive regions in the *Drosophila* blastoderm.. PLoS Biol.

[61] Wunderlich Z, Mirny LA (2009). Different gene regulation strategies revealed by analysis of binding motifs.. Trends Genet.

[62] Metchat A, Akerfelt M, Bierkamp C, Delsinne V, Sistonen L (2009). Mammalian heat shock factor 1 is essential for oocyte meiosis and directly regulates Hsp90alpha expression.. J Biol Chem.

[63] Chang Y, Ostling P, Akerfelt M, Trouillet D, Rallu M (2006). Role of heat-shock factor 2 in cerebral cortex formation and as a regulator of p35 expression.. Genes Dev.

[64] Zimarino V, Tsai C, Wu C (1990). Complex modes of heat shock factor activation.. Mol Cell Biol.

[65] Weinmann AS, Farnham PJ (2002). Identification of unknown target genes of human transcription factors using chromatin immunoprecipitation.. Methods.

[66] Ren B, Robert F, Wyrick JJ, Aparicio O, Jennings EG (2000). Genome-wide location and function of DNA binding proteins.. Science.

[67] Andres AJ, Thummel CS (1994). Methods for quantitative analysis of transcription in larvae and prepupae.. Methods in Cell Biology.

[68] Neal SJ, Gibson ML, So AK-C, Westwood JT (2003). Construction of a cDNA-based microarray for *Drosophila melanogaster*: A comparison of gene transcription profiles from SL2 and Kc167 cells.. Genome.

[69] Saeed AI, Sharov V, White J, Li J, Liang W (2003). TM4: A free, open-source system for microarray data management and analysis.. BioTechniques.

[70] Saeed AI, Bhagabati NK, Braisted JC, Liang W, Sharov V (2006). TM4 microarray software suite.. Methods Enzymol.

[71] Huang W, Sherman B, Lempicki R (2009). Systematic and integrative analysis of large gene lists using DAVID bioinformatics.. Nat Protoc 4.

[72] Workman CT, Yin Y, Corcoran DL, Ideker T, Stormo GD (2005). enoLOGOS: A versatile web tool for energy normalized sequence logos.. Nucleic Acids Res.

